# Chemical Constituents from *Coleus strobilifer* and Their Xanthine Oxidase Inhibitory Activity

**DOI:** 10.3390/molecules31010030

**Published:** 2025-12-22

**Authors:** Jia-Xu Qin, Yang Hong, Xiao-Na Gan, Ting-Zhao Li, Meng-Qi Wang, Xiang-Wei Zheng, Bo Li, Xin Fang, Shuang Liang

**Affiliations:** 1 Engineering Research Center of Modern Preparation Technology of Traditional Chinese Medicine, Ministry of Education, Innovation Research Institute of Traditional Chinese Medicine, Shanghai University of Traditional Chinese Medicine, Shanghai 201203, China; 2 Amway (Shanghai) Innovation & Science Co., Ltd., 720 Cailun Road, Shanghai 201203, China

**Keywords:** *Coleus strobilifer*, abietane diterpenoid, xanthine oxidase inhibitory activity

## Abstract

*Coleus strobilifer*, the dried rhizome and root of *Coleus strobilifer* (Roxb.) A.J. Paton, is widely used for dampness-detoxification and detumescence in Chinese folklore. This study marks the first comprehensive investigation into the chemical composition of the whole herb of *C. strobilifer*, leading to the isolation and identification of two new abietane diterpenes, 10*R*-carnosuain (**1**) and 10*R*-coleon U-3-one (**2**), along with 34 known compounds (**3**–**36**) isolated from *C. strobilifer* for the first time. Their structures were unambiguously elucidated by analyses of NMR, HRESIMS, IR, CD, and single-crystal X-ray diffraction data, and comparison with the literature. All the isolated compounds were screened for their xanthine oxidase (XO) inhibitory activity. Among them, apigenin (**8**), luteolin (**9**), and esculetin (**29**) showed moderate XO inhibitory activity with IC_50_ values of 0.034 ± 0.004, 0.067 ± 0.005, and 0.284 ± 0.01 mM, respectively.

## 1. Introduction

Worldwide, the prevalence of hyperuricemia and gout has increased dramatically due to excessive intake of high-purine foods and poor dietary habits, especially in economically developed regions such as Europe, America, East Asia, and Oceania [1]. Serum uric acid (SUA) concentration has been widely used as a diagnostic criterion for hyperuricemia [2]. Xanthine oxidase (XO) plays a key role in purine metabolism by directly catalyzing the conversion of hypoxanthine to xanthine and further to uric acid (UA), and the inhibition of XO could reduce the SUA level [3]. XO inhibitors are used in the treatment of hyperuricemia. Among the few therapeutic options, allopurinol and febuxostat have been the commonly used XO inhibitors in the clinic, but they could cause various adverse effects, such as allergy syndrome, impaired liver function and nephrotoxicity [1,2,3,4]. Hence, the discovery of natural XO inhibitors is particularly critical.

**Figure 1:**
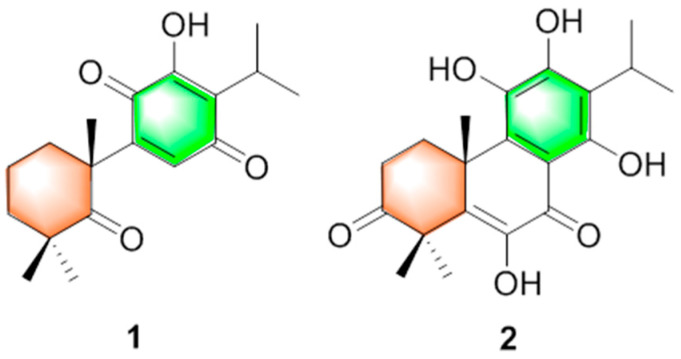
The chemical structures of compounds **1**–**2**.

The folk Chinese herbal medicine Xian-Pai-Cao is the dried rhizome and roots with old stems of *Coleus strobilifer* (Roxb.) A.J. Paton (syn. *Anisochilus carnosus* (L.f.) Wall. ex Benth), belonging to the Lamiaceae family, which is produced in Guangdong, Guangxi, and other places in Southern China, and also appears in India [5,6,7]. It has the efficacy of resolving dampness and removing turbidity, inducing diuresis and swelling, and is mainly used for treating summer-dampness, vomiting and diarrhea, edema, and urinary incontinence [8]. To date, there are limited studies on the phytochemical composition of *A. carnosua*, mainly focusing on the identification of common flavonoids, triterpenes, alkaloid, and essential oil [5,9,10,11]. Pharmacological studies show that crude extracts have antioxidant and anticancer activities [9,10,11,12]. Meanwhile, previous study found that 5 mg/mL of 70% ethanol extract of *C*. *strobilifer* possessed 99.97% of XO inhibition [13]. To explore the natural XO inhibitors from this plant, we conducted a systematic phytochemical investigation of *C*. *strobilifer* and screened the isolated compounds for XO inhibitory activity. As a result, two new abietane diterpenes (Figure 1), 10*R*-carnosuain (**1**) and 10*R*-coleon U-3-one (**2**), together with five known abietane diterpenes (**3**–**7**), three flavonoids (**8**–**10**), seven phenols (**11**–**17**), four sterols (**18**–**21**), four triterpenes (**22**–**25**), three sesquiterpenes (**26**–**28**), and nine other compounds (**29**–**36**) were obtained from the roots, stems and leaves of *C*. *strobilifer*. Furthermore, XO inhibitory activity of these isolates was evaluated. Herein, the isolation, structural elucidation, and XO inhibitory activity of these isolated compounds were described.

## 2. Results

**Figure 2:**
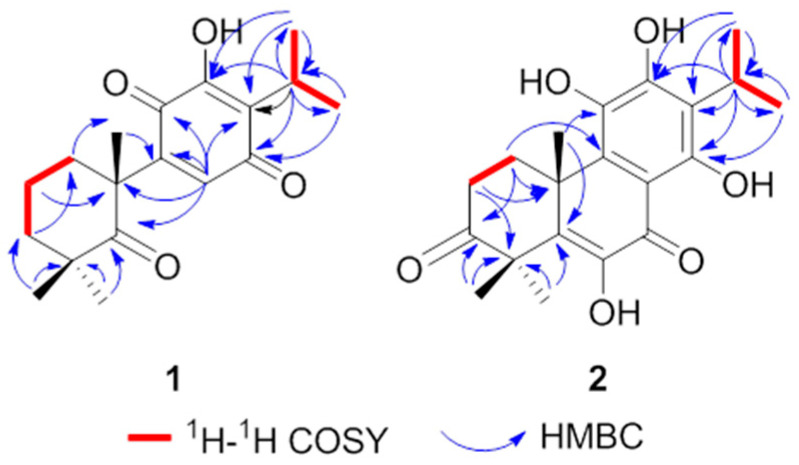
Key ^1^H-^1^H COSY and HMBC correlations of compounds **1**–**2**.

**Figure 3:**
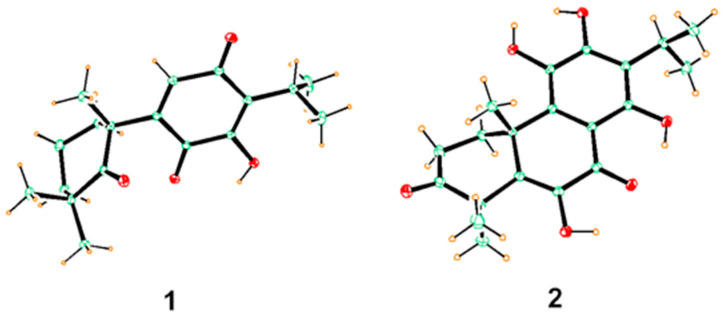
X-ray ORTEP drawing of compounds **1**–**2**.

Compound **1** was obtained as yellow needles crystals (MeOH). Its molecular formula was determined to be C_18_H_24_O_4_ by HRESIMS data (*m/z* 305.1753 [M + H] ^+^, calcd. for C_18_H_25_O_4_, 305.1747), corresponding to 7 degrees of unsaturation. The IR spectrum showed absorption bands for hydroxyl (3390 cm^−1^) and carboxyl groups (1651 cm^−1^, 1635 cm^−1^). The ^1^H NMR spectrum showed five methyls [*δ*_H_ 1.20 (6H, s), 1.21 (3H, s), 1.22 (3H, s), 1.38 (3H, s)], one olefinic methines [*δ*_H_ 6.51 (1H, s)] and one hydroxyl [*δ*_H_ 6.92 (s, 1H)] (Table 1). The ^13^C NMR and DEPT spectrum revealed 18 skeletal carbon signals including five methyls, three methylenes, two methines, five quaternary carbons (including one oxygenated and three olefinic ones) and three carbonyl carbons (*δ*_C_ 218.0, 187.3, 183.3) (Table 1). These spectroscopic features combined with HMBC correlations suggested that **1** was an abietane diterpene similar to royleanone [14], excepting the loss of two methylenes and one methine with an increase in one carbonyl group. The NMR data of **1** showed oxidative cleavage of C-5/6 and C-6/7 and formation a ketone (*δ*_C_ 218.0, C-5) compared to those of royleanone, which also could be confirmed by HRESIMS data and HMBC correlations from H-20 to C-5 and C-9, from H-8 to C-5, C-9, C-10, and from H-18/19 to C-5 (Figure 2). Unambiguously, the planar structure and 10*R* configuration of **1** were determined by single crystal X-ray diffraction analysis, and named 10*R*-carnosuain (Figure 3). Compound **1** was a structurally novel 6,7-norditerpenoid, for which spectroscopic and single-crystal data were reported for the first time in this study.

Compound **2** was isolated as yellow needles crystals (MeOH). The molecular formula was determined as C_20_H_24_O_6_ by HRESIMS data (*m/z* 361.1653 [M + H] ^+^, calcd. for C_20_H_25_O_6_, 361.1646). The ^1^H NMR spectrum showed the signals of five methyls [*δ*_H_ 1.34 (3H, s), 1.36 (3H, s), 1.42 (3H, s), 1.55 (6H, s)]. The ^13^C NMR data revealed 20 carbon signals including five methyls, two methylenes, one methine, eight olefinic carbons, two quaternary carbons, and three carbonyl carbons with the aid of DEPT and HMQC spectrum (Table 1). The above spectral data suggested that this compound was an abietane diterpenoid with an aromatic C-ring. The ^1^H-^1^H COSY established the fragments of C-1/C-2 (-CH_2_-CH_2_-) and C-15/C-16/17 (-CH-2CH_3_) (Figure 2). These features were similar to those of the coisolated coleon U (**5**) [15], except for the difference in the chemical shift in C-2 and C-4, suggesting the existence of a carbonyl carbon at C-3 (*δ*_C_ 217.7). Moreover, the HMBC correlations from H-1, H-2, H-18, and H-19 to C-3 and HRESIMS data again proved the conclusion. The final structure and 10R configurations of **2** were confirmed by single crystal X-ray diffraction analysis, and named 10*R*-coleon U-3-one (Figure 3).

Additionally, the 34 reported compounds (**3**–**36**) were confirmed by comparing their spectroscopic data with the literature data, and were identified as coleon U quinone (**3**) [15], 6,7-Dehydroroyleanone (**4**) [16], 5,6-Dihydrocoleon U (**5**) [17], coleon U (**6**) [15], 15-acetoxy-8,13*E*-labdadien-7-one (**7**) [18], apigenin (**8**) [19], luteolin (**9**) [19], rutin (**10**) [20], protocatechualdehyde (**11**) [21], caffeic acid (**12**) [19], caffeic acid ethyl ester (**13**) [22], caffeic acid ethylene ester (**14**) [19], salicylic acid (**15**) [21], carvacrol (**16**) [23], thymol (**17**) [23], β-sitosterol (**18**) [24], stigmasta-4,22-dien-3-one (**19**) [24], stigmasta- 4,22-dien-3,6-dione (**20**) [25], stigmasta-5,22-dien-3,7-dione (**21**) [26], ursolic acid (**22**) [27], ursolic aldehyde (**23**) [27], maslinic acid (**24**) [21], tormentic acid (**25**) [21], α-cedrene (**26**) [28], clovane-2,9-diol (**27**) [24], α-cyperone (**28**) [29], esculetin (**29**) [19], gusanlung C (**30**) [30], (1*R*,2*R*,2′*E*)-2-[5′-(hydroxy)-2-penten-1-yl]-3-oxocyclopentane acetic acid methyl ester (**31**) [31], vomifoliol (**32**) [32], m-cymene (**33**) [33], 5-hydroxymethylfurfural (**34**) [32], cineole (**35**) [34], and azelaic acid (**36**) [35]. Notably, all the known compounds were identified in *C. strobilifer* for the first time. The inhibitory effects of all isolated compounds were evaluated against XO. In the assay system, allopurine, which showed IC_50_ values of 0.009 mM, was used as positive control. Compounds **8**, **9**, and **29** exhibited moderate inhibition on XO with IC_50_ values of 0.034 ± 0.004, 0.067 ± 0.005 and 0.284 ± 0.01 mM, respectively (Table S1 in Supplementary Information).

## 3. Discussion

Up to the present, the chemical composition of *C. strobilifer* remains largely unexplored, and previous biological studies have been confined to crude extracts [9,10,11,12]. Our work provides the first systematic analysis of its chemical profile and, for the first time, screens the isolated pure compounds for XO inhibitory activity, offering new insights into the bioactive principles of this plant.

As a result, 36 compounds were isolated from *C. strobilifer*, including two new abietane diterpenes, 10*R*-carnosuain (**1**), and 10*R*-coleon U-3-one (**2**), together with 34 known compounds (**3**–**36**), which were isolated from *C. strobilifer* for the first time. These include seven abietane diterpenoids (**1**–**7**), three flavonoids or flavonoid glycosides (**8**–**10**), seven phenolic compounds (**11**–**17**), eight sterols (**18**–**25**), three sesquiterpenes (**26**–**28**), and eight other types of constituents (**29**–**36**). It was worth mentioning that compound **1** was a structurally novel 6,7-norditerpenoid, for which spectroscopic and single-crystal data were reported firstly.

The chemical profile of *C. strobilifer* is mainly dominated by abietane-type diterpenoids, consistent with phytochemical trends in the genus Coleus, which is known for its rich diterpenoid diversity, with over 240 analogues reported to date [36]. The discovery of a rare 6,7-norditerpenoid not only expands the structural repertoire of the genus but may also serve as a species-specific chemotaxonomic marker [37]. While most abietanes in Coleus conform to classical skeleton types such as royleanones, spirocoleons, and quinone derivatives, compound 1 deviates from these frameworks, suggesting that *C. strobilifer* may harbour unique enzymatic modifications shaping its chemical profile.

In addition to abietane diterpenoids, the remaining metabolites, including flavonoids, phenolics, sterols, sesquiterpenoids, and various other compounds, underscore the structural and functional diversity of *C. strobilifer*. Flavonoids and phenolics are likely key contributors to redox-related bioactivities, such as antioxidant, anti-inflammatory, and antibacterial effects [38,39]. Sterols, as essential components of plant cell membranes, play critical roles in cellular physiology, development, and stress adaptation [40]. Although less abundant, sesquiterpenes may mediate ecological interactions, including antimicrobial defence and allelopathic functions [41]. The additional miscellaneous metabolites further demonstrate the extensive metabolic versatility of *C. strobilifer*, highlighting a complex biochemical repertoire that extends far beyond its predominant diterpenoid constituents.

All isolated compounds were initially screened for XO inhibition at 200 μM. Only compounds **8**, **9**, **29**, and the *C. strobilifer* extract exhibited notable activity (Figure S19). The IC_50_ values of compounds **8**, **9**, and **29** were determined as 0.034 ± 0.004, 0.067 ± 0.005, and 0.284 ± 0.01 mM, respectively, showing moderate inhibition compared to the positive control (0.009 mM) (Figure S20 and Table S1). To evaluate whether these compounds were the key active constituents responsible for the extract’s activity, their contents in both aqueous and alcoholic extracts of *C. strobilifer* were analyzed using UPLC. Further investigation revealed that, at *C. strobilifer* extract concentration of 1 mg/mL, compounds **8**, **9**, and **29** reached maximum levels of 0.397, 0.207, and 0.645 μg/mL, respectively (Figure S21 and Table S3). Despite the C. strobilifer alcohol extract exhibiting 90.1% XO inhibition at this concentration, the individual compounds or their combination accounted for less than 11.0% inhibition (Table S4). These results suggest that other constituents or potential synergistic interactions contribute significantly to the overall XO inhibitory activity of the extract, highlighting the complexity of its bioactive profile. These findings clearly indicate that the three compounds contribute minimally to the observed XO inhibitory activity and cannot fully represent the material basis of *C. strobilifer* extracts. Therefore, while this work significantly expands the phytochemical knowledge of *C. strobilifer*, the principal bioactive components underlying its pronounced XO inhibitory activity remain to be identified and warrant further investigation.

## 4. Materials and Methods

### 4.1. General Experimental Procedures

One-dimensional (^1^H and ^13^C) NMR and two-dimensional (^1^H–^1^H COSY, HSQC, HMBC, and NOESY) NMR experiments were performed on an AVANCE NEO 400 or 600 MHz spectrometer (Bruker, Fällanden, Switzerland) operating at 400 or 600 MHz for ^1^H NMR and 101 or 151 MHz for ^13^C NMR, respectively. Chemical shifts were expressed in *δ* (ppm) and coupling constants in Hz. The ^1^H NMR spectra were collected with 16 repetitions, while the ^13^C NMR spectra were acquired with 1024 co-added scans. HRESIMS data were acquired using an Agilent 6545 Q-TOF LC/MS (Agilent, Palo Alto, CA, USA). The IR spectrum was obtained from a Shimadzu IRAffinity-1S spectrometer (Shimadzu Corporation, Kyoto, Japan) using KBr pellets, with a resolution of 4 cm^−1^ and 8 scans co-added. Optical rotations were measured by a Perkin Elmer Model 341 polarimeter (Perkin-Elmer, Waltham, MA, USA). X-ray structure was determined on a Bruker D8 venture X-ray diffractometer (Bruker, Billerica, MA, USA). Semi-preparative HPLC was performed on a Shimadzu LC-6AD system with a C18 column (21.2 × 250 mm, 7 μm). A mixture of acetonitrile-H_2_O was used as eluent. The following materials were utilized: YMC-Gel ODS-A-HG (YMC Co. Ltd., Kyoto, Japan); silica gel (200–300 mesh; Jiangyou Silica Gel Development Co., Ltd., Yantai, China); Sephadex LH-20 (GE Chemical Corporation, Waupaca, WI, USA).

### 4.2. Plant Material

*C. strobilifer* was collected in November 2022 from Zhangzhou city, Fujian Province, China, and authenticated by Associate Professor Zhaohui Xu of Shanghai University of Traditional Chinese Medicine. A voucher specimen (BCY102901) was deposited at Shanghai University of Traditional Chinese Medicine.

### 4.3. Extraction and Isolation

The dried plant (20 kg) was extracted with 80% EtOH (3 × 80 L) under reflux, giving an extraction yield of 9.87%. The extract (1974.89 g) was fractionated via chromatography to afford **1**–**36** (see Supplementary Information, isolation of all compounds).

#### 4.3.1. 10*R*-Carnosuain

Yellow, needles crystals (MeOH); ${\left[\alpha \right]}_{D}^{25}$ −162.08 (*c* 0.25, MeOH); IR (KBr) *γ*_max_ 2927, 2870, 1697, 1651, 1635, 1612, 1379, 1288, 1004 cm^−1^; ^1^H and ^13^C NMR data are shown in Table 1; HRESIMS (*m/z*) 305.1754 [M + H] ^+^ (calcd for C_18_H_25_O_4_, 305.1757).

#### 4.3.2. 10*R*-Coleon U-3-One

Yellow, needles crystals (MeOH); ${\left[\alpha \right]}_{D}^{25}$ +21.16 (*c* 0.5, MeOH); IR (KBr) *γ*_max_ 3385, 2960, 2927, 1701, 1597, 1448, 1340, 1303, 1251, 968 cm^−1^; ^1^H and ^13^C NMR data are shown in Table 1; HRESIMS (*m/z*) 3 61.1653 [M + H] ^+^ (calcd for C_20_H_25_O_6_, 361.1646).

#### 4.3.3. X-Ray Crystal Structure Analysis

Suitable crystals of compounds **1** and **2** were both obtained at room temperature from MeOH solutions. The crystallographic data of **1** and **2** were deposited at the CCDC (numbers 2417597 and 2417599, respectively)

Crystal data for **1**: C_54_H_72_O_12_, M = 913.11, a = 6.43390 (10) Å, b = 24.1490 (5) Å, c = 16.5007 (4) Å, *α* = 90°, *β* = 94.6670 (10)°, *γ* = 90°, V = 2555.25 (9) Å^3^, T = 299 (2) K, space group P 1 21 1, Z = 2, *µ* (cu k*α*) = 0.669 mm^−1^, 72,851 reflections collected, 8752 independent reflections [R (int) = 0.1100], the final R1 indices were 0.0582 (*I* > 2*σ* (*I*)), the final wR2 indices were 0.1433 (*I* > 2*σ* (*I*)), the final R1 indices were 0.0881 (all data), the final wR2 indices were 0.1675 (all data), the goodness-of-fit on F^2^ was 1.015.

Crystal data for **2**: C_20_H_24_O_6_, M = 360.39, a = 8.366 (2) Å, b = 13.326 (5) Å, c = 16.353 (4) Å, *α* = 90°, *β* = 90°, *γ* = 90°, V = 2555.25 (9) Å^3^, T = 299 (2) K, Space group P 21 21 21, Z = 4, *µ* (cu k*α*) = 0.798 mm^−1^, 51023 reflections collected, 3126 independent reflections [R (int) = 0.0868], the final R1 indices were 0.0373 (*I* > 2*σ* (*I*)), the final wR2 indices were 0.0961 (*I* > 2*σ* (*I*)), the final R1 indices were 0.0401 (all data), the final wR2 indices were 0.0981 (all data), the goodness-of-fit on F2 was 1.054.

### 4.4. Determination of Xanthine Oxidase Inhibitory Activity

The XO inhibition assay of all compounds was assessed according to the method reported in the literature with minor modification [42] (see Supplementary Information, xanthine oxidase inhibition assay).

## 5. Conclusions

This study presents the first comprehensive phytochemical investigation of *Coleus strobilifer* and provides new insights into the bioactive constituents responsible for its traditional medicinal use. A total of 36 compounds were isolated, including two new abietane diterpenes, 10*R*-carnosuain (**1**) and 10*R*-coleon U-3-one (**2**), along with 34 known compounds, all reported from this species for the first time. Structural elucidation was achieved through extensive spectroscopic analysis, and the absolute configurations of the two new diterpenes were confirmed by single-crystal X-ray diffraction. Among the isolates, compounds **8**, **9**, and **29** exhibited moderate XO inhibitory activity. However, further analysis showed that their concentrations within the crude extract at 1 mg/mL were very low, and their individual or combined inhibitory effects accounted for only a small fraction of the strong XO inhibition previously reported for the extract. These findings indicate that the major active constituents responsible for the potent anti-hyperuricemic potential of *C. strobilifer* remain unidentified.

## Figures and Tables

**Table 1:** ^1^H (400 MHz) and ^13^C NMR (101 MHz) spectral data of compounds **1** and **2**.

No.	Compound 1 ^1^		Compound 2 ^2^	
	*δ*_C_ (ppm)	*δ*_H_ (ppm, *J* in Hz)	*δ*_C_ (ppm)	*δ*_H_ (ppm, *J* in Hz)
1	38.8	1.59 (dq, *J* = 12.7, 2.8 Hz, 1H) 2.02, overlap	28.6	3.45 (1H, m) 1.88 (1H, dt, *J* = 13.7, 9.8 Hz)
2	18.4	1.92, overlap 1.76, overlap	34.2	2.80 (1H, ddd, *J* = 19.0, 9.3, 1.4 Hz) 2.64 (1H, ddd, *J* = 19.0, 10.5, 9.1 Hz)
3	38.8	1.97, overlap 1.73, overlap	217.7	
4	44.7		50.0	
5	218.0		140.5	
6			142.1	
7			182.8	
8	134.7	6.51 (1H, s, H-8)	106.9	
9	150.1		135.5	
10	49.9		40.2	
11	183.3		136.7	
12	151.2		154.4	
13	125.5		121.1	
14	187.3		159.4	
15	34.1	3.16 (1H, hept, *J* = 7.1 Hz, H-13)	26.0	3.48 (1H, m, H-15)
16	19.9	1.20 (3H, s, H-14)	20.5	1.34 (3H, s, H-16)
17	19.9	1.20 (3H, s, H-15)	20.6	1.36 (3H, s, H-17)
18	28.0	1.22 (3H, s, H-16)	21.6	1.55 (3H, s, H-18)
19	28.4	1.21 (3H, s, H-17)	25.1	1.55 (3H, s, H-19)
20	23.4	1.38 (3H, s, H-18)	21.6	1.42 (3H, s, H-20)

^1^ Measured in CDCl_3_; ^2^ Measured in CD_3_OD.

## Data Availability

The data underlying this article are available in the article and Supplementary Information.

## Supplementary Materials

The following supporting information can be downloaded at: https://www.mdpi.com/article/10.3390/molecules31010030/s1, Isolation of all compounds; Xanthine oxidase inhibition assay; Figure S1. Chemical structures of compounds **1**–**20**; Figure S2. Chemical structures of compounds **21**–**36**; Figure S3–S18. 1D NMR, 2D NMR, IR and HR-ESI-MS spectrum of compounds **1** and **2**; Spectral data of compounds **3**–**36**; Figure S19. Xanthine oxidase inhibitory activity of compounds **1**–**36** and *C. strobilifer* extract; Figure S20. XO inhibitory activity of compounds **8**, **9**, **29** and allopurinol. Table S1. XO inhibitory activity of compounds **1**–**36** and allopurinol; Determination of the content of compounds **8**, **9**, and **29** in *Coleus strobilifer*; Table S2. Analytical figures of merit by UPLC; Figure S21. UPLC of standards and samples; Table S3. The contents of **8**, **9**, and **29** in *C. strobilifer* extract; Table S4. XO inhibitory activity of compounds **8**, **9**, **29**, and *C. strobilifer* extract.
