# Supplementary material for: Chemical Constituents from Coleus strobilifer and Their Xanthine Oxidase Inhibitory Activity
Source: Molecules. 2025 Dec 22;31(1):30. doi: 10.3390/molecules31010030 (PMC12786449; doi:10.3390/molecules31010030)
Supplement: Supplementary file 1 [file molecules-31-00030-s001.zip › molecules-3984024-supplementary.pdf]

Supplementary Information

Chemical constituents from *Coleus strobilifer* and their xanthine oxidase inhibitory activity

Jia-Xu Qin [1,†], Yang Hong [1,†], Xiao-Na Gan [2], Ting-Zhao Li [2], Meng-Qi Wang [1], Xiang-Wei Zheng [1], Bo Li [2,*], Xin Fang [1,*] and Shuang Liang [1,*]

[1] Engineering Research Center of Modern Preparation Technology of Traditional Chinese Medicine, Ministry of Education, Innovation Research Institute of Traditional Chinese Medicine, Shanghai University of Traditional Chinese Medicine, Shanghai 201203, China; qinjiaxu2020@163.com (J.-X.Q.); 15805651024@163.com (Y.H.); 15668436335@163.com (M.-Q.W.); zhengxwsh@hotmail.com (X.-W.Z.)

[2] Amway (Shanghai) Innovation & Science Co., Ltd., 720 Cailun Road, Shanghai 201203, China; shana.gan@amway.com (X.-N.G.); teric.li@amway.com (T.-Z.L.)

* Corresponding author's address: Shanghai University of Traditional Chinese Medicine, Shanghai, China. Tel./Fax: +86 21 51322429/+86 21 51322491 (X.F.; S.L.)

E-mail: robert.li@amway.com (B. L.); felix.fx@163.com (Xin Fang); ls7312@163.com (Shuang Liang).

[†] These authors contributed equally to this work.

*Coleus strobilife*r, the dried rhizome and root of *Coleus strobilifer* (Roxb.) A. J. Paton, is widely used for dampness-detoxification and detumescence in Chinese folklore. This study marks the first comprehensive investigation into the chemical composition of the whole herb of *C. strobilifer*, leading to the isolation and identification of two new abietane diterpenes, 10*R*-carnosuain (**1**) and 10*R*-coleon U-3-one (**2**), along with 34 known compounds (**3-36**). Their structures were unambiguously elucidated by analyses of NMR, HRESIMS, UV, IR and single-crystal X-ray diffraction data, and comparison with literature. All the isolated compounds were screened for their xanthine oxidase (XO) inhibitory activity. Among them, apigenin (**8**), luteolin (**9**) and esculetin (**29**) showed moderate XO inhibitory activity with IC_{50} values of 0.034 ± 0.004, 0.067 ± 0.005 and 0.284 ± 0.01 mM, respectively.

Keywords: *Coleus strobilifer*; abietane diterpenes； xanthine oxidaseinhibitory activity

Contents

Isolation of all compounds....4

Xanthine oxidase inhibition assay....6

Figure S1 Chemical structures of compounds **1-20**....7

Figure S2 Chemical structures of compounds **21-36**....8

Figure S3 ^{1}H NMR (400 MHz, $CDCl_3$) spectrum of compound **1**....9

Figure S4 ^{13}C NMR (101 MHz, $CDCl_3$) spectrum of compound **1**....9

Figure S5 ^{1}H-^{1}H COSY spectrum of compound **1** in $CDCl_3$....10

Figure S6 HMQC spectrum of compound **1** in $CDCl_3$....10

Figure S7 NOESY spectrum of compound **1** in $CDCl_3$....11

Figure S8 HMBC spectrum of compound **1** in $CDCl_3$....11

Figure S9 IR (KBr) spectrum of compound **1** 12

Figure S10 HR-ESI-MS spectrum of compound **1**....12

Figure S11 ^{1}H NMR (400 MHz, CD_3OD) spectrum of compound **2**....13

Figure S12 ^{13}C NMR (101 MHz, CD_3OD) spectrum of compound **2**....13

Figure S13 ^{1}H-^{1}H COSY spectrum of compound **2** in CD_3OD....14

Figure S14 HMQC spectrum of compound **2** in CD_3OD....14

Figure S15 NOESY spectrum of compound **2** in CD_3OD....15

Figure S16 HMBC spectrum of compound **2** in CD_3OD....15

Figure S17 IR (KBr) spectrum of compound **2**....16

Figure S18 HR-ESI-MS spectrum of compound **2**....16

Spectral data of compounds **3-36**....16

Figure S19 XO inhibitory activity of compounds **1-36** and *C. strobilifer*....25

Figure S20 XO inhibitory activity of compounds **8**, **9**, and **29** and allopurinol....25

Table S1 Xanthine oxidase inhibitory activity of compounds **1-36** and allopurinol...25

Determination of the content of compounds **8**, **9**, and **29** in *Anisochilus carnosus*.....25

Table S2 Analytical figures of merit by UPLC....26

Figure S21 UPLC of standards and samples (A- standard solution, B- sample solution,

1- esculetin (**29**), 2- luteolin (**9**), 3- apigenin (**8**)). 26
Table S3 The contents of **8**, **9**, and **29** in *C. strobilifer* extract. 27
Table S4 XO inhibitory activity of compounds **8**, **9**, **29**, and *A. carnosus* extract.......27

Isolation of all compounds

The EtOH extracts (1974.9 g) of *Coleus strobilifer* were separated by silica gel column chromatography and eluted successively with CH_2Cl_2-MeOH (100:0-5:1) to give Fr-A- Fr-E according to TLC analysis. Fr-A (80.2 g) was separated by repeated silica gel CC and eluted successively with PE-EtOAc (1:0-0:1) to obtain Fr-A1- Fr-A3. Fr-A1 (12.6 g) was separated by repeated silica gel CC and eluted successively with PE- CH_2Cl_2 (1:0-0:1) to obtain Fr-A1a- Fr-A1c; Fr-A1a (2.1 g) was purified by Sephadex LH-20 (MeOH: CH_2Cl_2, 1:1) and was then purified by preparative TLC (PTLC) to obtain compound **26** (11 mg) and **27** (7 mg); Fr-A1b (1.7 g) was purified by Sephadex LH-20 (MeOH: CH_2Cl_2, 1:1) and was then purified by re-crystallization to obtain compound **28** (11 mg) and **35** (23 mg); Fr-A1c (2.8 g) was purified by Sephadex LH-20 (MeOH: CH_2Cl_2, 1:1) to obtain compound **33** (17 mg). Fr-A2 (16.7 g) was separated by repeated silica gel CC and eluted successively with PE- EtOAc (100:0-10:1) to afford Fr-A2a- Fr-A2c, and was then by Sephadex LH-20 (MeOH: CH_2Cl_2, 1:1) and recrystallization to obtain compounds **6** (283 mg), **18** (31 mg), **19** (26 mg), **20** (32 mg) and **21** (21 mg), respectively. Fr-A3 (15.3 g) was separated by repeated silica gel CC and eluted successively with PE- EtOAc (1:0-5:1) to afford Fr-A3a- Fr-A3d; Fr-A3a (4.5 g) was purified by Sephadex LH-20 (MeOH: CH_2Cl_2,

1:1) and recrystallization to obtain compound **4** (2.2 g); Fr-A3b (1.8 g) was purified by semi-preparative HPLC (30% ACN-70% H_2O) to give compound **2** (28 mg); Fr-A3c (3.1 g) was purified by semi-preparative HPLC (77% ACN-23% H_2O) to give compound **3** (230 mg); Fr-A3d (0.9 g) was purified by LH-20 (MeOH: CH_2Cl_2, 1:1) to give compound **7** (3 mg). Fr-B (19 g) was purified by ODS CC (MeOH: H_2O, 30%-90%) to afford compounds **16** (45 mg), **22** (7 mg), **23** (28 mg) and **27** (29 mg). Fr-C (12 g) was separated by repeated silica gel CC and eluted successively with CH_2Cl_2-MeOH (80:0-10:1, in 0.1% HCOOH) to obtain Fr-C1- Fr-C3; Fr-C1 (1.9 g) was purified by ODS CC (MeOH: H_2O, 50%- 30%, in 0.1% HCOOH) to afford compounds **23** (28 mg) and **24** (37 mg); Fr-C2 (1.8 g) was purified by LH-20 (MeOH: CH_2Cl_2, 1:1) to give compound **29** (17 mg). Fr-D (10 g) was separated by ODS CC (MeOH: H_2O, 30%- 90%) and was then purified by LH-20 (MeOH) to obtain compounds **9** (37 mg), **10** (24 mg), **15** (17 mg), and **34** (25 mg). Fr-E (75 g) was separated by repeated silica gel CC and eluted successively with CH_2Cl_2-MeOH (50:0-5:1) to obtain Fr-E1- Fr-E3; Fr-E1 (3.5 g) was purified by recrystallization to obtain compound **1** (450 mg), and was then purified by PTLC to obtain compound **5** (48 mg). Fr-E2 (5.3 g)was separated by ODS CC (MeOH: H_2O, 30%- 90%) to produce Fr-E2a- Fr-E2d; Fr-E2a (2.1 g) purified by LH-20 (MeOH) to obtain compounds **8** (47 mg) and **32** (18 mg); Fr-E2b (0.9 g) was purified by LH-20 (MeOH) to obtain compounds **11** (18 mg) and **12** (35 mg); Fr-E2c (1.1 g) was purified by ODS CC (MeOH: H_2O, 50%- 80%) to obtain compounds **31** (47 mg) and **36** (6 mg); Fr-E3

(4.7 g) was separated by LH-20 (MeOH) to produce Fr-E3a and Fr-E3b; Fr-E3a (1.8 g) was purified by ODS CC (MeOH: H_2O, 30%- 70%) to obtain compounds **13** (28 mg) and **14** (39 mg); Fr-E3b (1.1 g) was purified by repeated silica gel CC and eluted successively with CH_2Cl_2-MeOH (30:1) to obtain compound **30** (18 mg).

Xanthine oxidase inhibition assay

The reaction mixture included 50 μL of the sample and 50 μL of 0.02 U/mL, XO solution that was properly shaken for 30 s and incubated at 37 ℃ for 5 min. Then, 150 μL of 0.48 mM xanthine solution was added, and again, the mixture was carefully shaken for 30 s and incubated at 37 ℃ for 30 min. Finally, the absorbance at 290 nm was measured, and the inhibitory activity was calculated using the following equation:

$$\textit{Inhibition percentage}\ (\%) = [1 - (A_1\text{-}A_2)/(A_3\text{-}A_4)]\times 100$$

where A_1 is the absorbance of the production with the sample and XOD, A_2 is the absorbance of the production with the sample, A_3 is the absorbance of the production with the buffer solution and XOD, and A_4 is the absorbance of the production with the buffer solution. Each experiment was repeated three times. Allopurinol was used as a positive control. The extent of inhibition and IC_{50} values were calculated by GraphPad Prism 6.0 software.

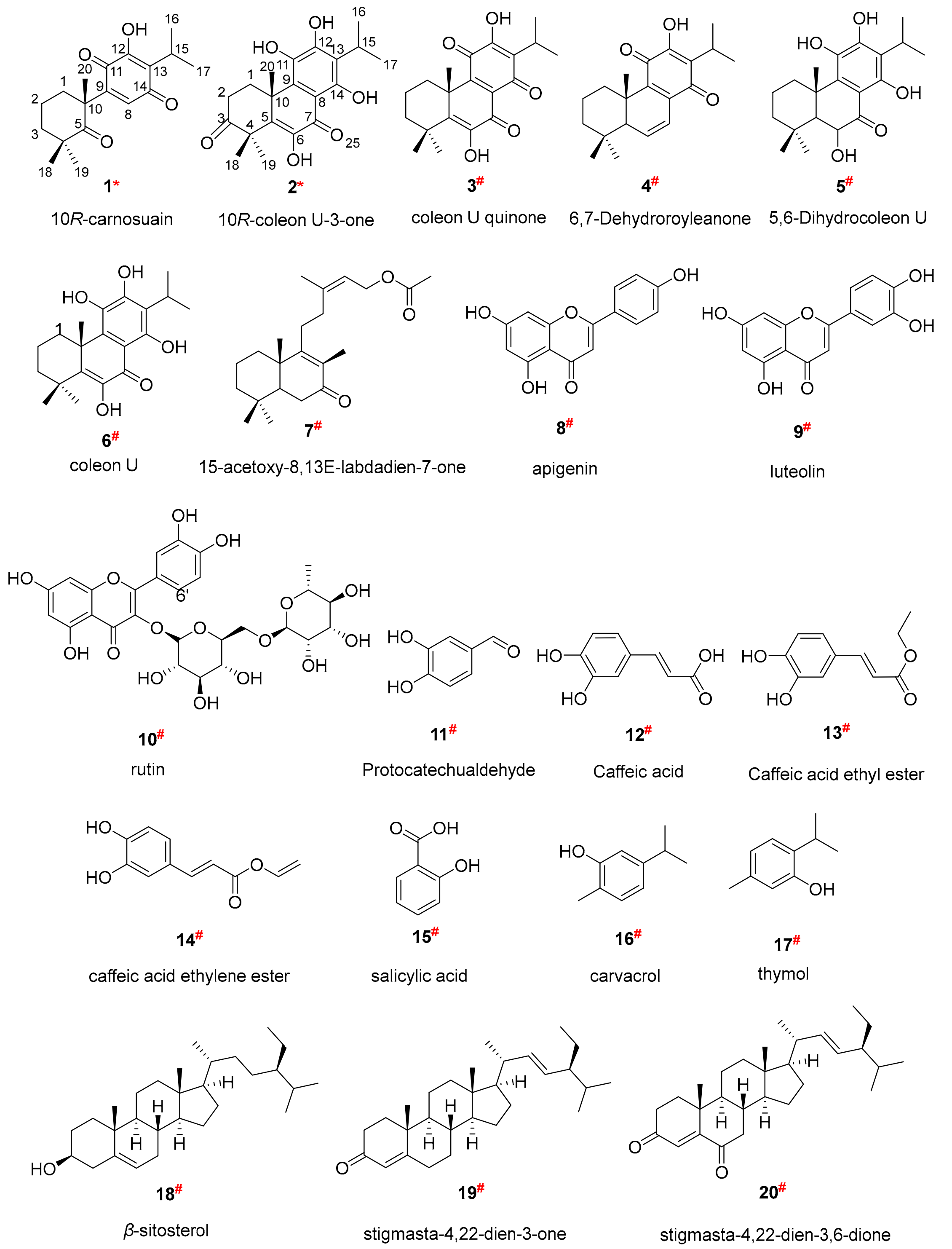

Figure S1 Chemical structures of compounds **1-20**.

* New compounds were isolated from *Coleus strobilifer*. # Compounds have not been reported in *Coleus strobilifer*.

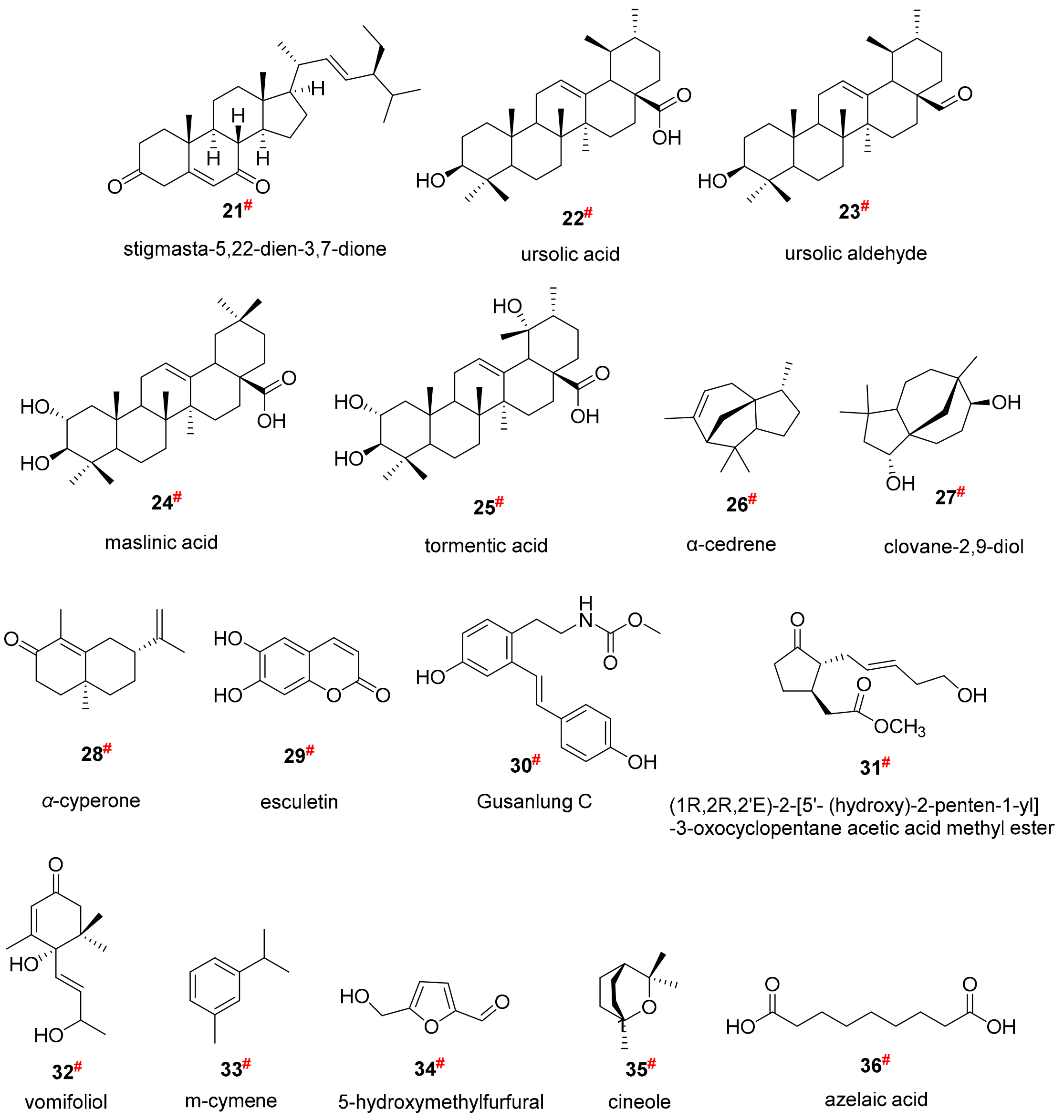

Figure S2 Chemical structures of compounds **21**-**36**.

Compounds have not been reported in *Coleus strobilife*r.

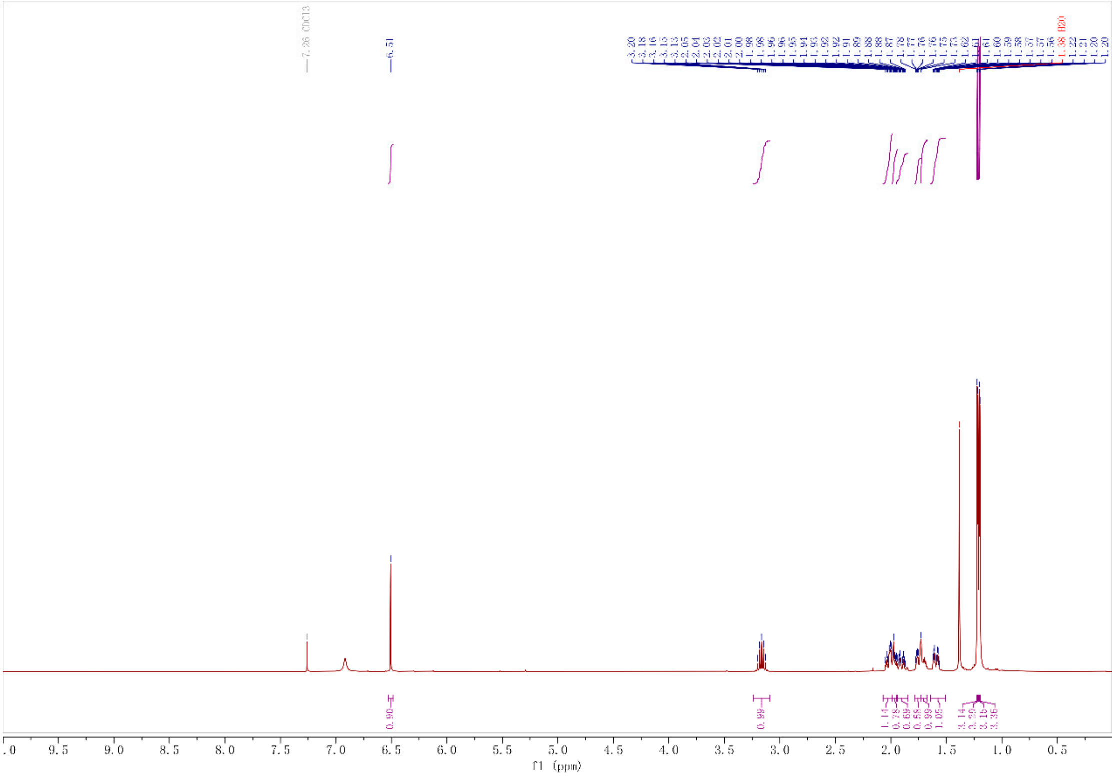

Figure S3 ^{1}H NMR (400 MHz, $CDCl_3$) spectrum of compound **1**.

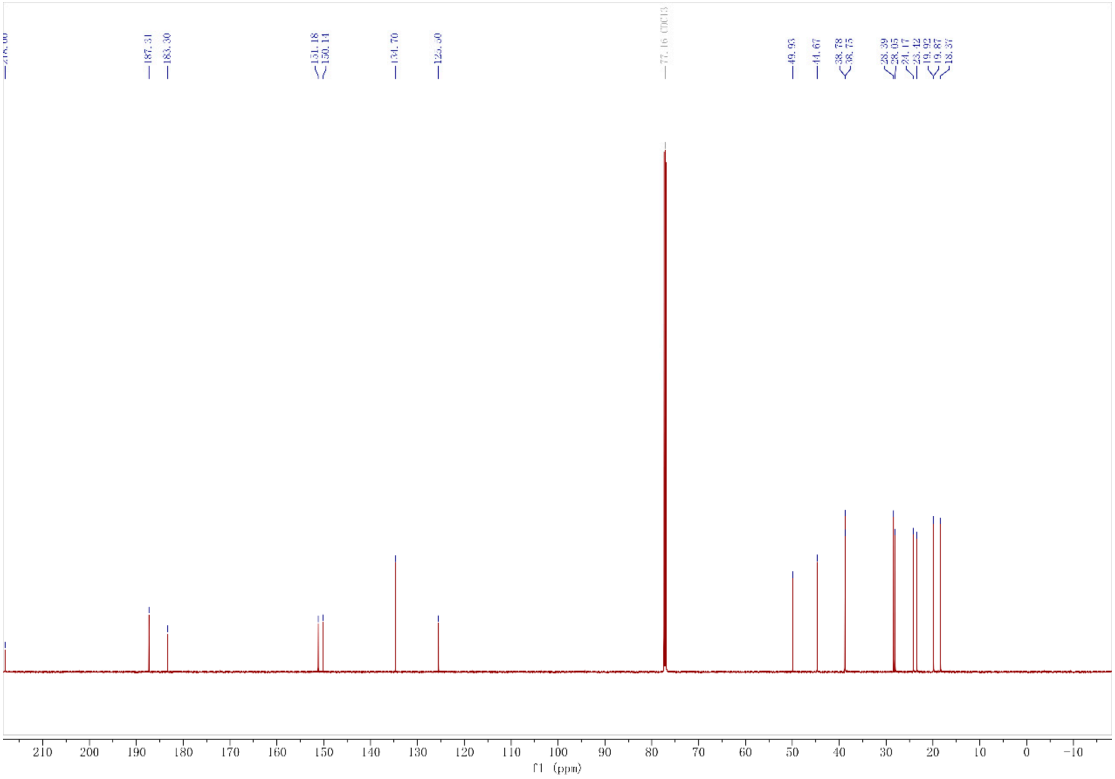

Figure S4 ^{13}C NMR (101 MHz, $CDCl_3$) spectrum of compound **1**.

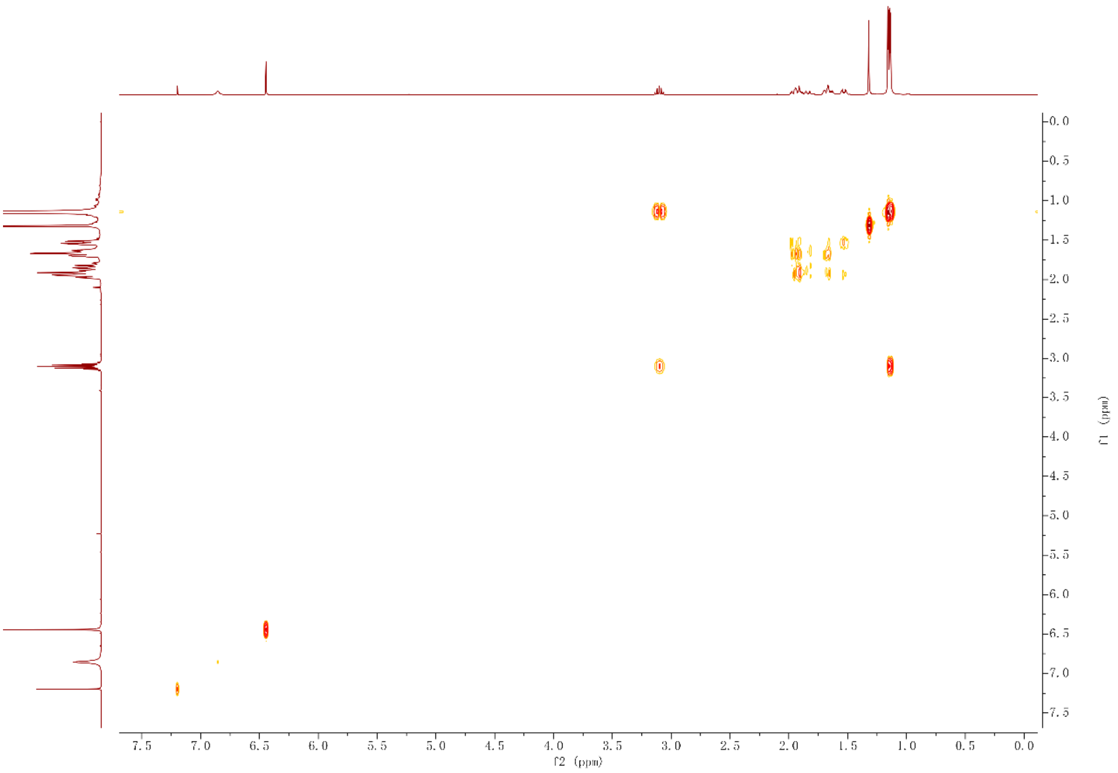

Figure S5 ^{1}H-^{1}H COSY spectrum of compound **1** in $CDCl_3$.

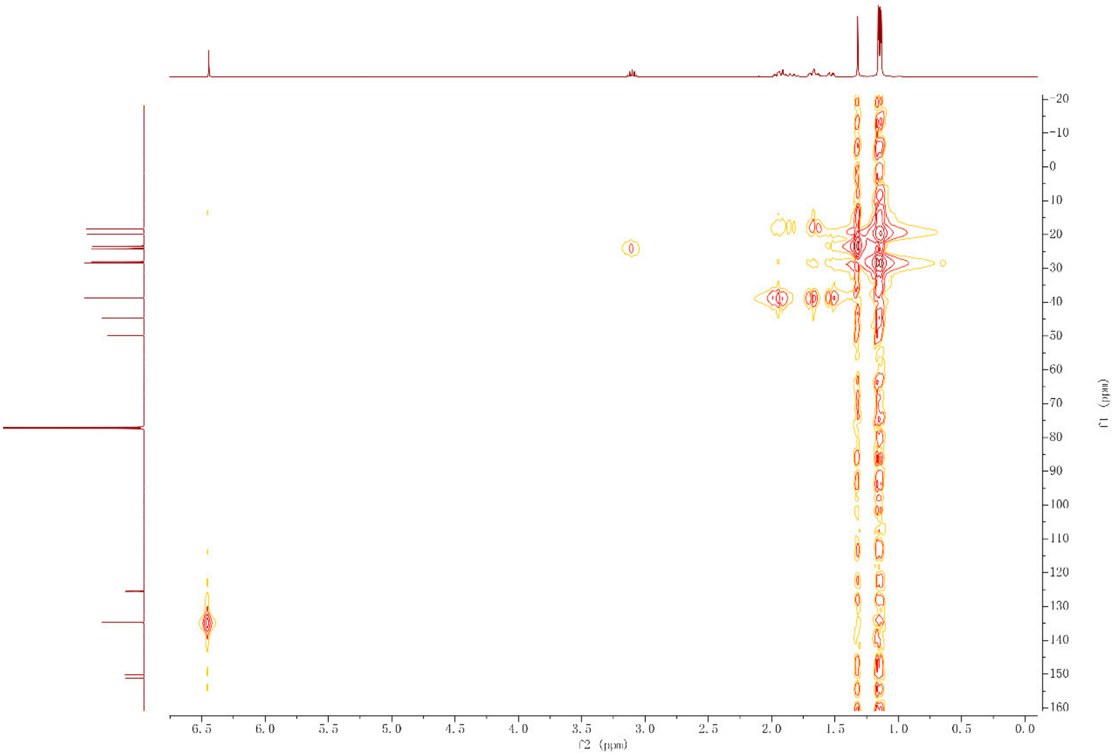

Figure S6 HMQC spectrum of compound **1** in $CDCl_3$.

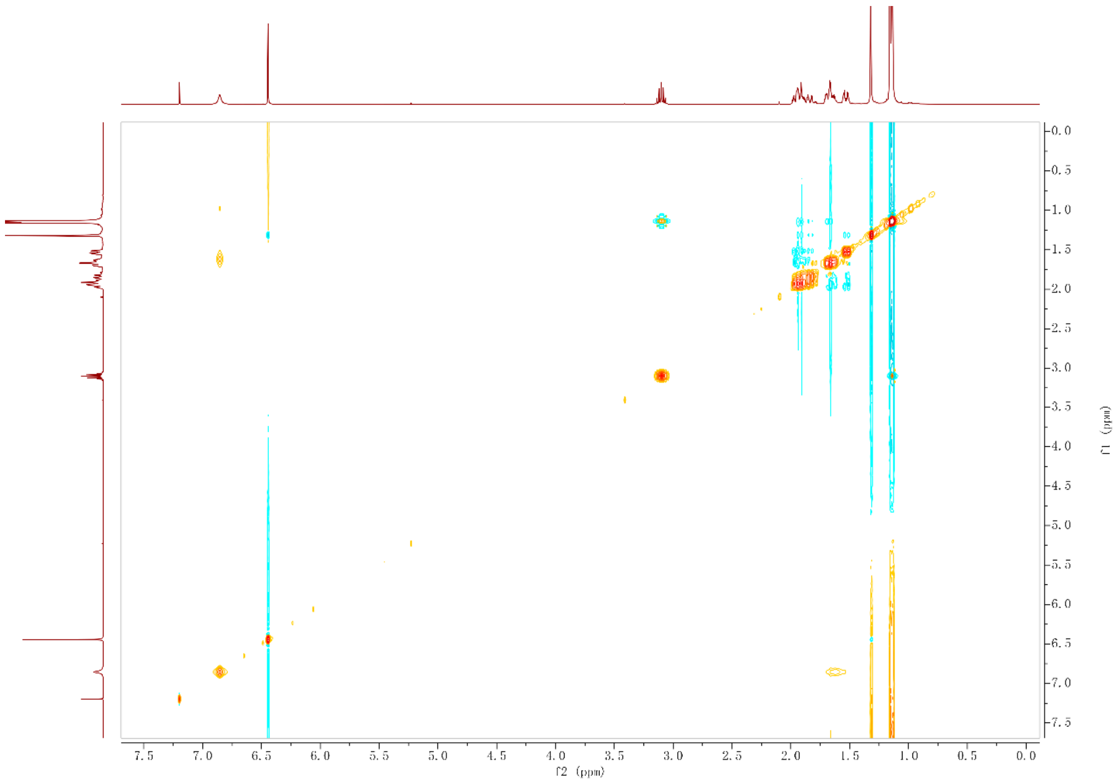

Figure S7 NOESY spectrum of compound **1** in $CDCl_3$.

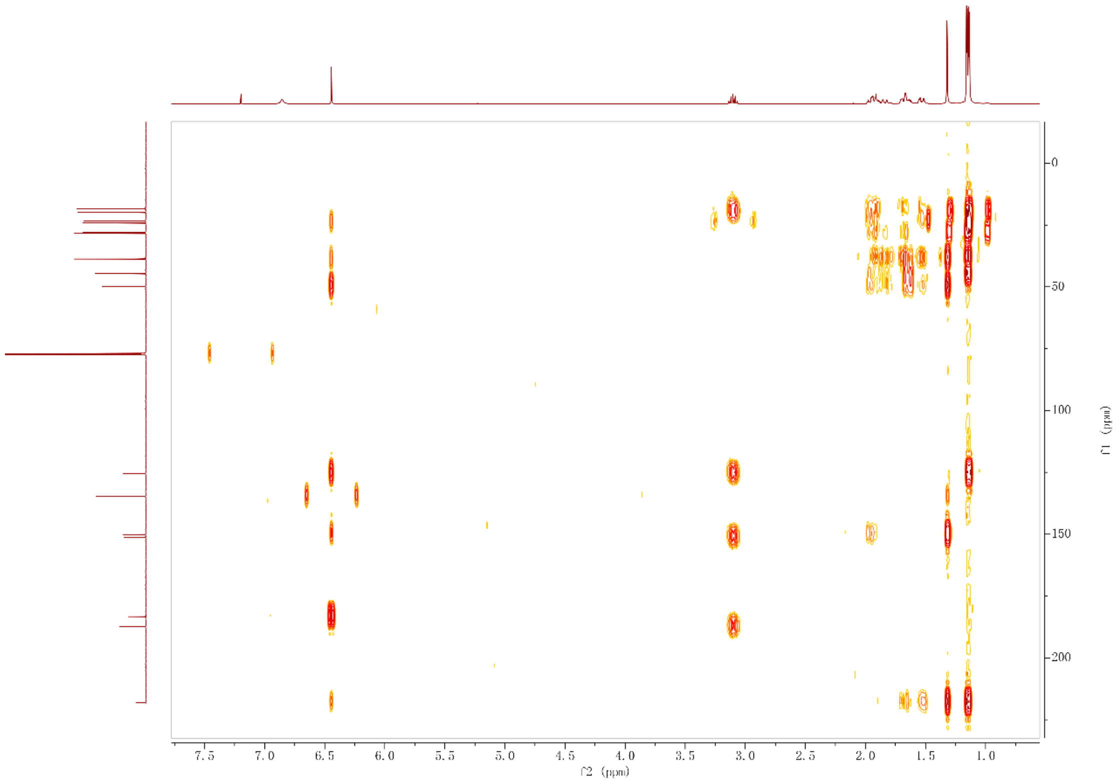

Figure S8 HMBC spectrum of compound **1** in $CDCl_3$.

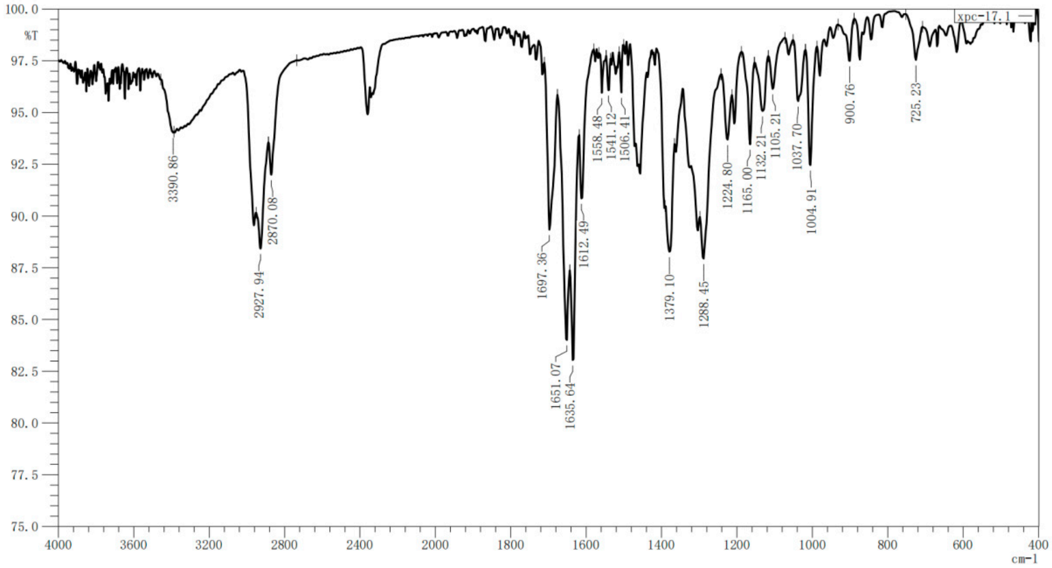

Figure S9 IR (KBr) spectrum of compound **1**.

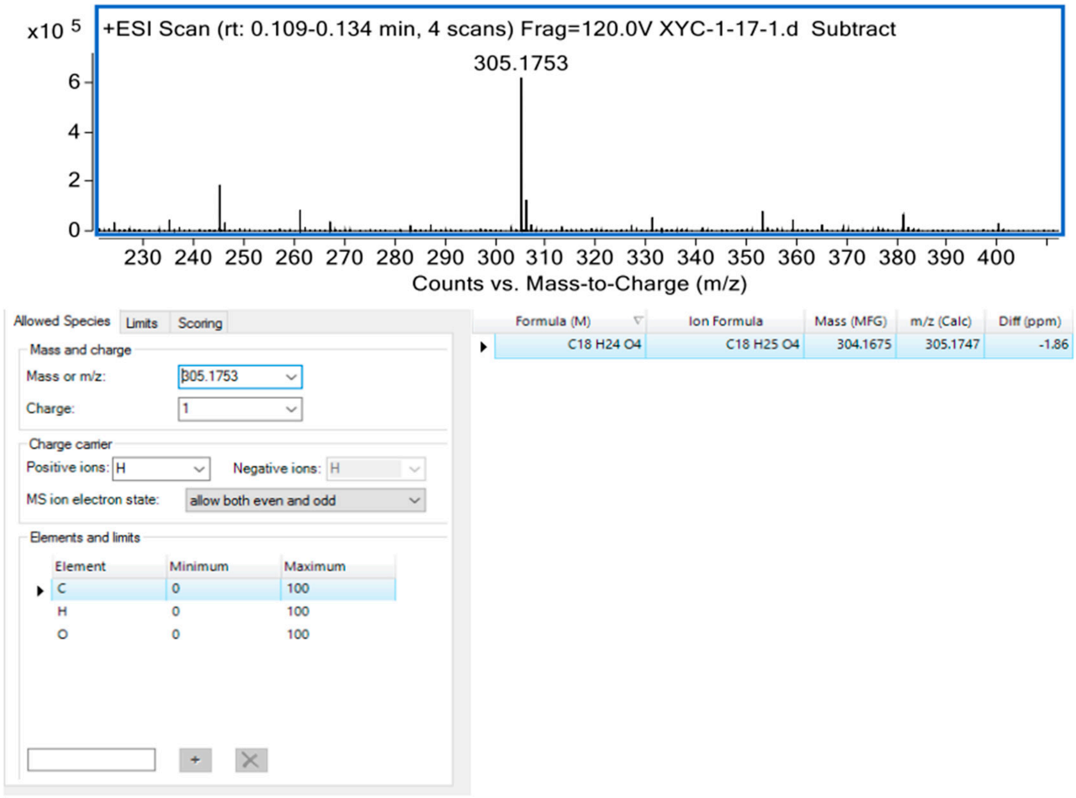

Figure S10 HR-ESI-MS spectrum of compound **1**.

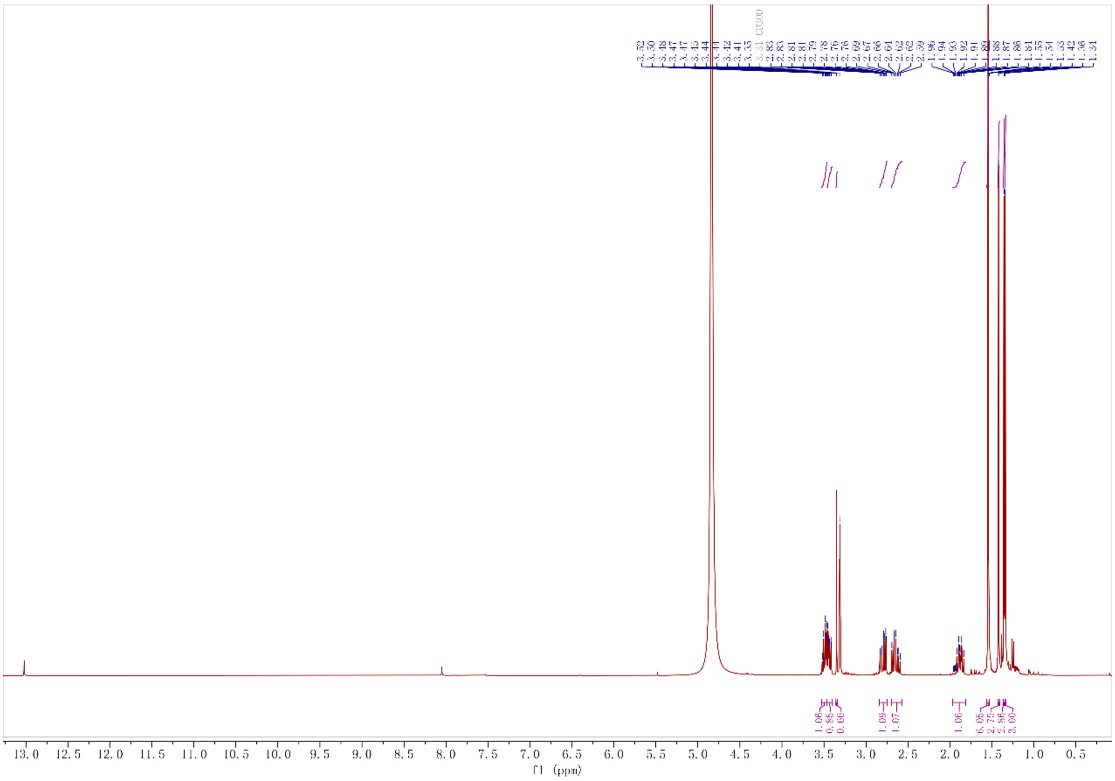

Figure S11 ^{1}H NMR (400 MHz, CD_3OD) spectrum of compound **2**.

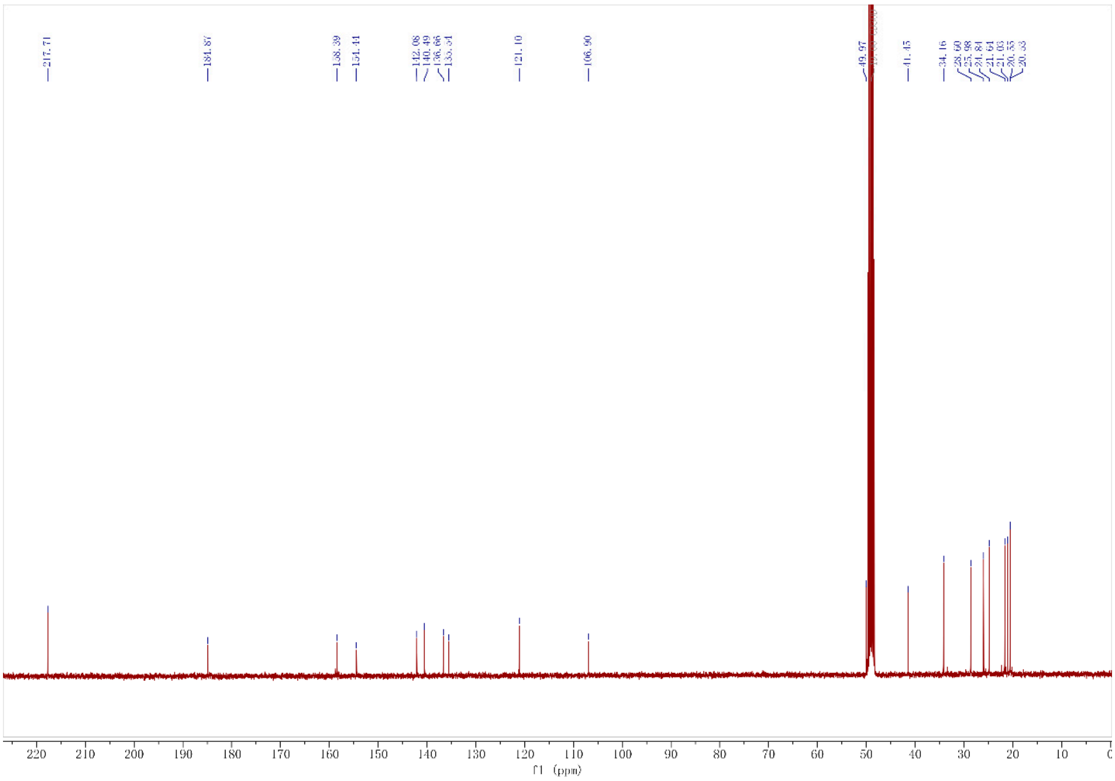

Figure S12 ^{13}C NMR (101 MHz, CD_3OD) spectrum of compound **2**.

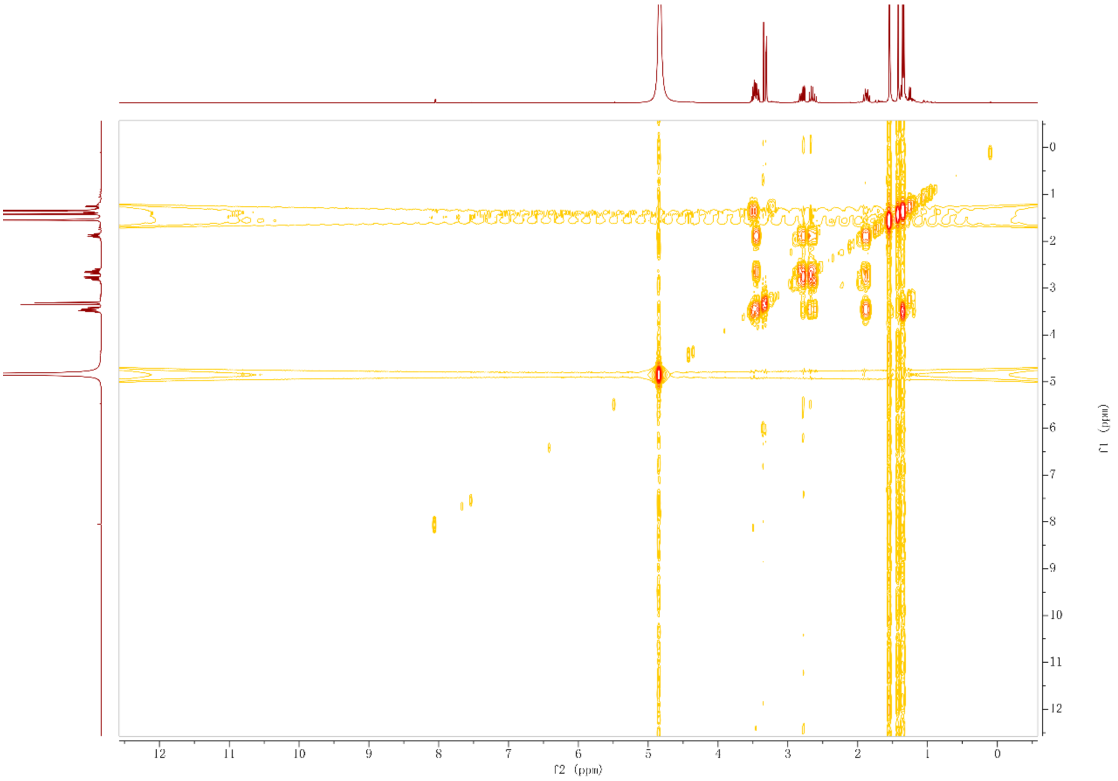

Figure S13 ^{1}H-^{1}H COSY spectrum of compound **2** in CD3OD.

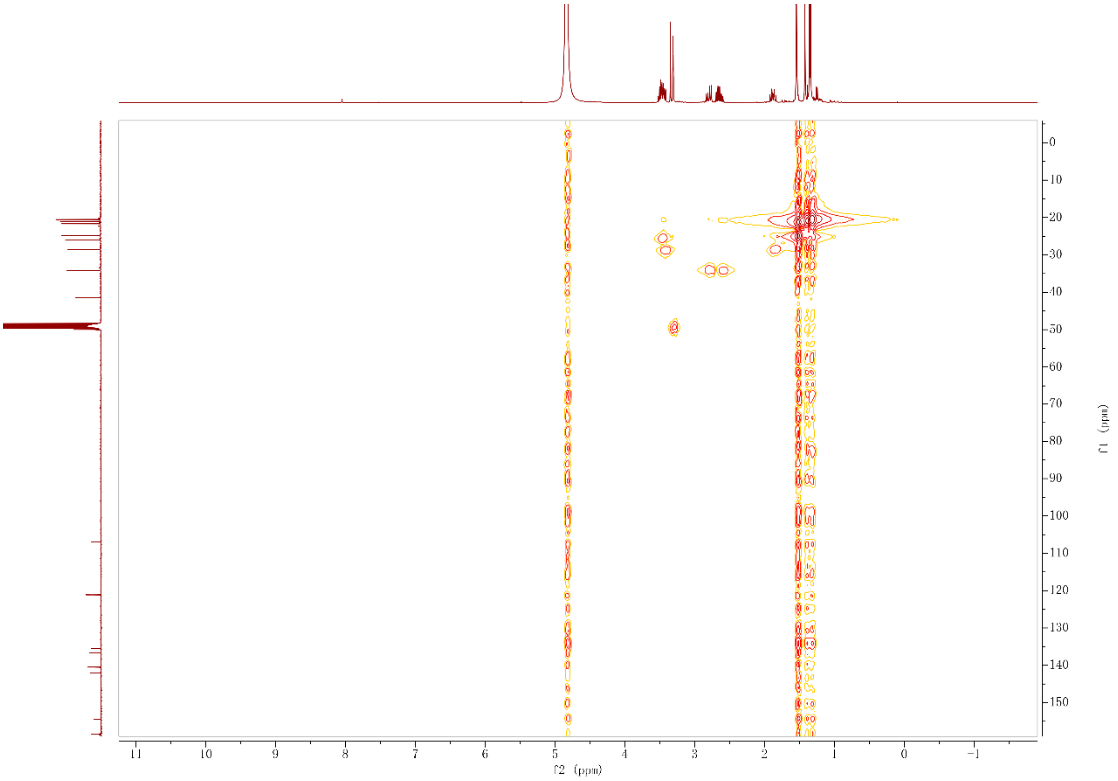

Figure S14 HMQC spectrum of compound **2** in CD3OD.

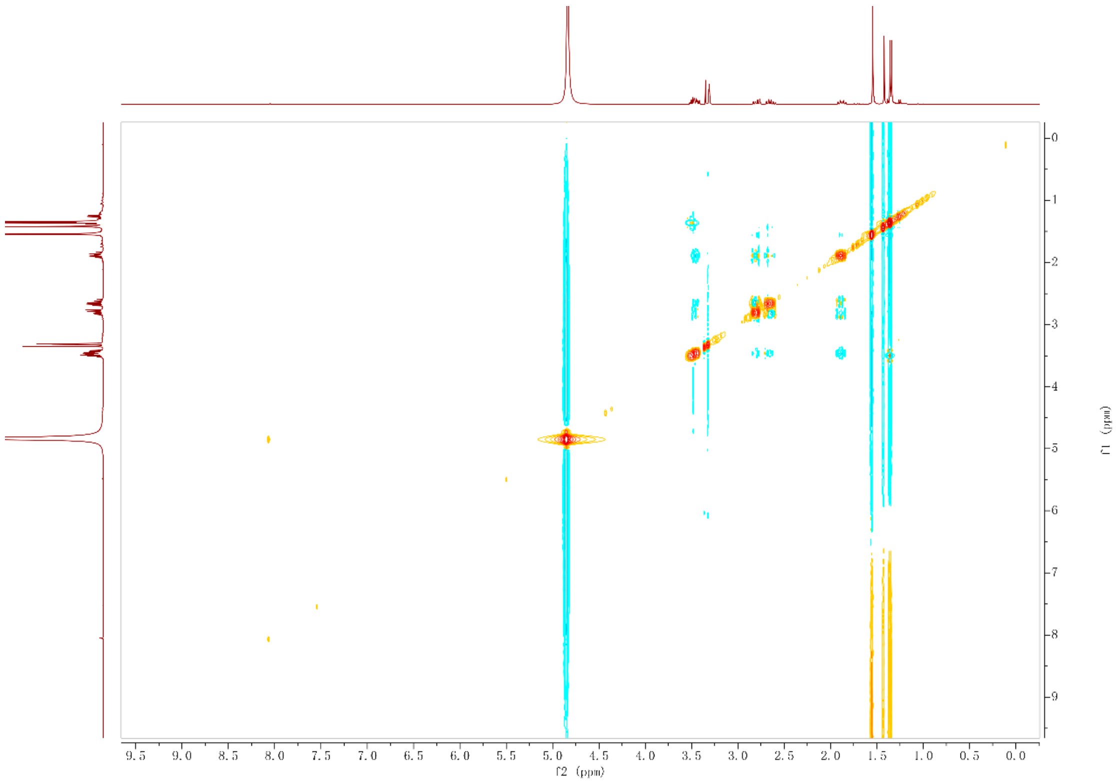

Figure S15 NOESY spectrum of compound **2** in CD_3OD.

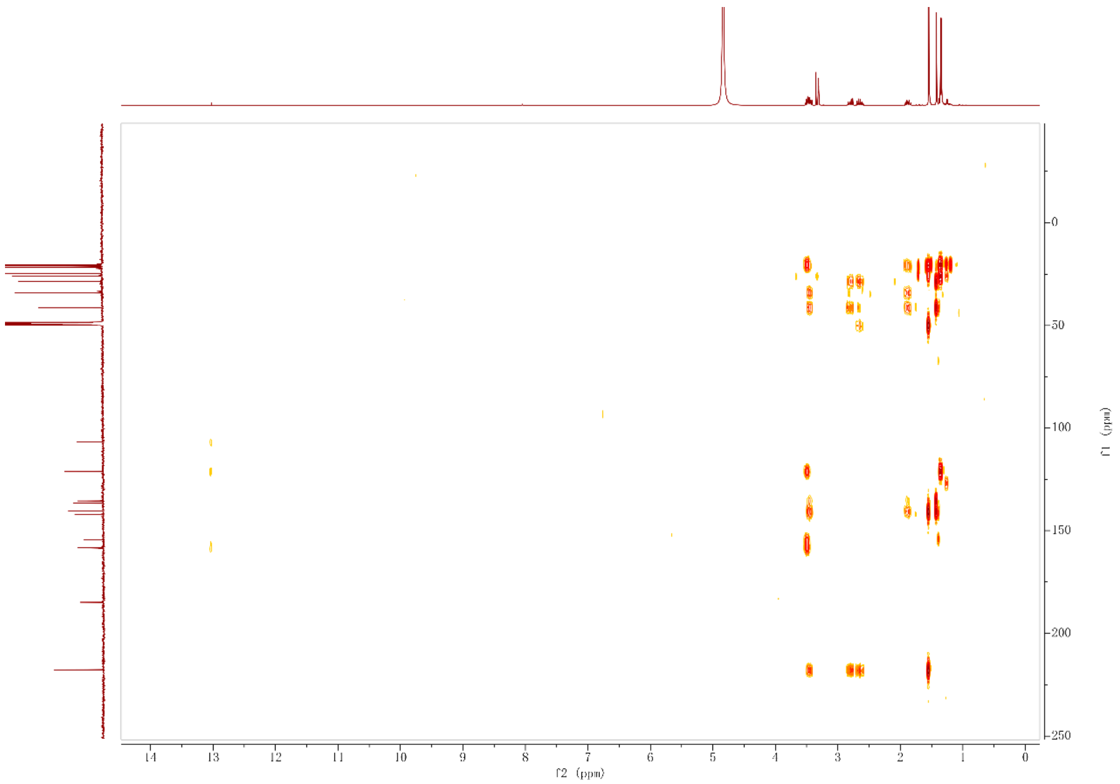

Figure S16 HMBC spectrum of compound **2** in CD_3OD.

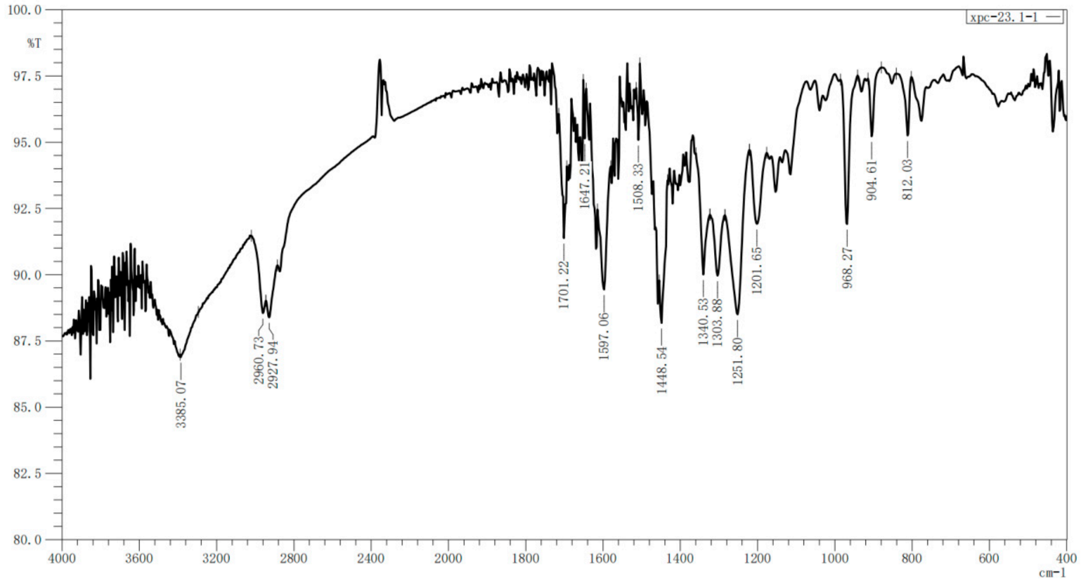

Figure S17 IR (KBr) spectrum of compound **2**.

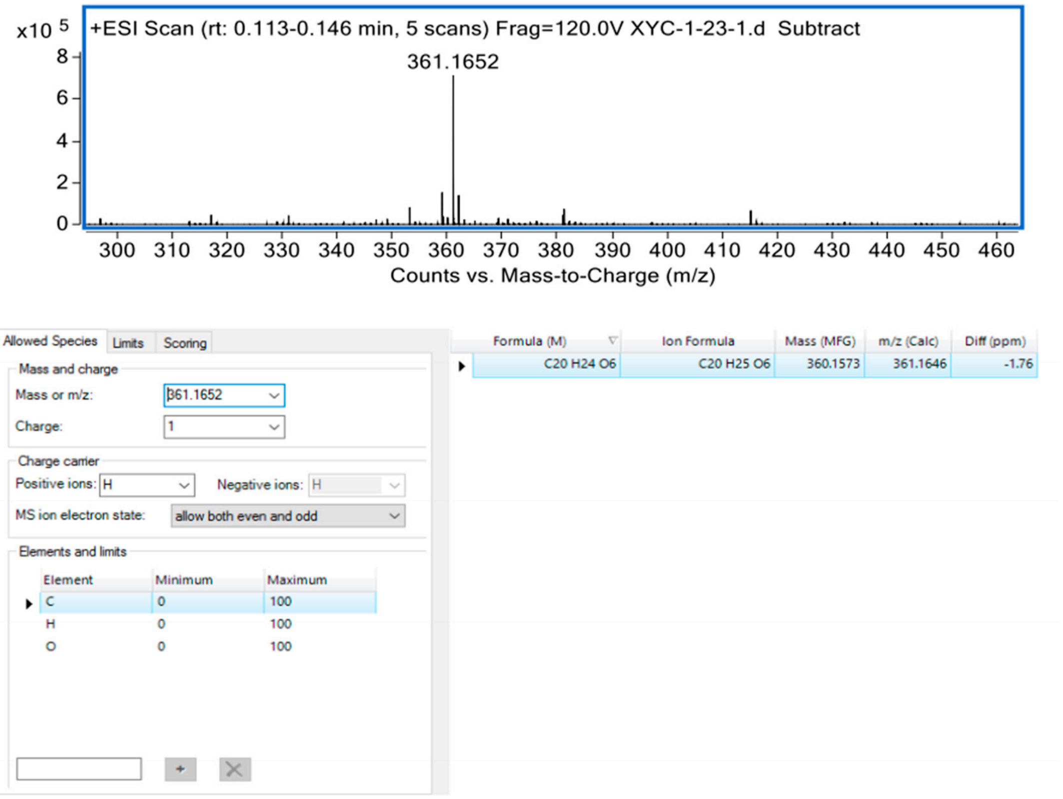

Figure S18 HR-ESI-MS spectrum of compound **2**.

Spectral data of compounds 3-36

coleon U quinone (**3**): Yellow powder; *m/z* 345.1692 $[M + H]^+$ (calcd for $C_{20}H_{27}O_3$, 315.1955). ^{1}H NMR (600 MHz, $CDCl_3$): δ_H 3.24 (1H, hept, *J* = 7.0 Hz, H-15), 1.26 (6H, d, *J* = 7.1,1.1 Hz, H-16/17), 1.44 (6H, d, *J* = 6.1 Hz, H-18/19), 1.64 (3H, s, H-20); ^{13}C NMR (151 MHz, $CDCl_3$): δ_C 30.9 (C-1), 17.8(C-2), 36.4 (C-3), 36.4 (C-4), 143.5 (C-5), 146.9 (C-6), 177.7 (C-7), 126.9 (C-8), 155.2 (C-9), 41.5 (C-10), 183.7 (C-11), 150.8 (C-12), 126.1 (C-13), 184.5 (C-14), 24.5 (C-15), 19.9 (C-16), 19.9 (C-17), 27.3 (C-18), 29.2 (C-19), 27.6 (C-20).

6,7-Dehydroroyleanone (**4**):Red needle-like crystals; *m/z* 315.1592 $[M + H]^+$ (calcd for $C_{20}H_{25}O_5$, 315.1955). ^{1}H NMR (600 MHz, $CDCl_3$): δ_H 2.92 (2H, d, *J* = 13.5 Hz, H-1), 1.57 (2H, m, H-2), 1.45 (2H, dd, *J* = 34.6, 13.2 Hz, H-3), 2.14 (1H, d, *J* = 3.4 Hz, H-5), 6.48 (1H, d, *J* = 9.7 Hz, H-6), 6.74 (1H, d, *J* = 9.6 Hz, H-7), 3.15 (1H, m, H-15), 1.18 (3H, d, *J* = 4.0 Hz, H-16), 1.20 (3H, d, *J* = 3.2 Hz, H-17), 0.98 (3H, d, *J* = 2.9 Hz, H-18), 1.03 (3H, s, H-19), 1.04 (3H, s, H-20); ^{13}C NMR (151 MHz, $CDCl_3$): δ_C 36.3 (C-1), 19.7 (C-2), 41.7 (C-3), 34.2 (C-4), 53.4 (C-5), 139.6 (C-6), 121.9 (C-7), 138.3 (C-8), 142.6 (C-9), 40.6 (C-10), 184.6 (C-11), 123.8 (C-13), 187.6 (C-14), 25.2 (C-15), 20.2 (C-16), 20.4 (C-17), 33.1 (C-18), 23.2 (C-19), 15.4 (C-20).

5,6-Dihydrocoleon U (**5**): Yellow powder; *m/z* 349.2007 $[M + H]^+$ (calcd for $C_{20}H_{29}O_5$, 349.2010).^{1}H NMR (400 MHz, $CDCl_3$): δ_H 1.63 (1H, m, H-5), 4.52 (1H, d, *J* = 2.5 Hz, H-6),3.44 (1H, d, *J* = 8.6 Hz, H-15), 1.33 (6H, s, H-16/17), 1.05 (3H, s, H-18), 1.34 (3H, s, H-19), 1.72 (3H, s, H-20); ^{13}C NMR (101 MHz, $CDCl_3$): δ_C 38.9 (C-1), 19.4 (C-2), 42.9 (C-3), 33.42 (C-4), 52.7 (C-5), 71.8 (C-6), 201.0 (C-7), 106.6 (C-8), 139.0 (C-9), 39.9 (C-10), 133.3 (C-11), 153.6 (C-12), , 118.8 (C-13), 161.3 (C-14), 24.5 (C-15), 20.3 (C-16), 20.4 (C-17), 24.3 (C-18), 35.9 (C-19), 22.6 (C-20).

coleon U (**6**): Yellow needle-like crystals; *m/z* 347.1860 $[M + H]^+$ (calcd for $C_{20}H_{27}O_5$, 347.1853). ^{1}H NMR (400 MHz, CD_3OD): δ_H 2.91 (1H, ddd, *J* = 14.3, 12.4, 6.3 Hz, H-15), 1.44 (3H, s, H-20), 1.22 (6H, d, *J* = 11.0 Hz, H-18, 19), 1.18 (6H, dd, *J* = 7.1, 1.9 Hz, H-16/17); ^{13}C NMR (101 MHz, CD_3OD): δ_C 18.7 (C-2), 20.5 (C-17), 20.6 (C-16), 25.6 (C-15), 27.4 (C-18), 28.4 (C-20), 28.6 (C-1), 31.5 (C-19), 37.2 (C-3), 37.3 (C-4), 42.2 (C-10), 106.1 (C-8), 120.0 (C-13), 135.3 (C-11), 140.1 (C-9), 142.9 (C-5), 144.1 (C-6), 154.2 (C-12), 157.9 (C-14), 184.1 (C-7).

15-acetoxy-8,13E-labdadien-7-one (**7**): White powder; *m/z* 369.2411 $[M + Na]^+$ (calcd for $C_{20}H_{34}NaO_3$, 369.2400). ^{1}H NMR (400 MHz, $CDCl_3$): δ_H 5.40 (1H, tq, *J* = 7.1, 1.3

Hz, H-14), 4.60 (2H, d, J = 7.0 Hz, H-15), 2.07 (s, 3H, -$COCH_3$), 1.76 (6H, d, J = 2.0 Hz, H-16, 17), 1.08 (3H, s, H-20), 0.92 (3H, s, H-19), 0.89 (3H, s, H-18); ^{13}C NMR (101 MHz, $CDCl_3$): δ_C 36.0 (C-1), 18.8 (C-2), 41.5 (C-3), 33.3 (C-4), 50.4 (C-5), 38.4 (C-6), 200.4 (C-7), 130.4 (C-8), 167.5 (C-9), 41.1 (C-10), 28.3 (C-11), 35.4 (C-12), 141.6 (C-13), 118.9 (C-14), 61.4 (C-15), 16.7 (C-16), 18.3 (C-17), 32.7 (C-18), 21.5 (C-19), 11.6 (C-20), 21.2 (-$COCH_3$), 171.2 (-$COCH_3$).

apigenin (**8**): Green powder; *m/z* 271.0599 $[M + H]^+$ (calcd for $C_{15}H_{11}O_5$, 271.0601). ^1H NMR (400 MHz, DMSO-d_6) δ_H 6.77 (1H, s, H-3), 6.19 (1H, d, J = 2.0 Hz, H-6), 6.48 (1H, d, J = 2.0 Hz, H-8), 7.92 (2H, d, J = 8.5 Hz, H-2', 6'), 6.92 (2H, d, J = 8.5 Hz, H-3', 5'); ^{13}C NMR (101 MHz, DMSO): δ_C 164.0 (C-2), 102.8 (C-3),181.7 (C-4), 161.2 (C-5),98.8 (C-6), 163.7 (C-7), 94.0 (C-8), 157.3 (C-9), 103.7 (C-10), 121.2 (C-1'), 128.5 (C-2'), 116.0 (C-3'), 161.5 (C-4'), 116.0 (C-5'), 128.5 (C-6').

luteolin (**9**): Yellow powder; *m/z* 287.0558 $[M + H]^+$ (calcd for $C_{15}H_{11}O_6$, 287.0550)。 ^1H NMR (400 MHz, CD_3OD): δ_H 7.41-7.35 (2H, m, H-2', H-6'), 6.90 (1H, d, J = 9.0 Hz, H-5'), 6.53 (1H, s, H-3), 6.44 (1H, d, J = 2.1 Hz, H-8), 6.21 (1H, d, J = 2.1 Hz, H-6); ^{13}C NMR (101 MHz, CD_3OD): δ_C 95.0 (C-8), 100.1 (C-6), 103.9 (C-3), 105.3 (C-10), 114.2 (C-2'), 116.8 (C-5'), 120.3 (C-1'), 123.7 (C-6'), 147.0 (C-3'), 151.0 (C-4'), 159.4 (C-9), 163.2 (C-5), 166.0 (C-2), 166.4 (C-7), 183.9 (C-4).

rutin (**10**): Yellow powder; *m/z* 633.1434 $[M + Na]^+$ (calcd for $C_{27}H_{31}O_{16}$, 633.1461). ^1H NMR (400 MHz, CD_3OD): δ_H 7.69 (1H, d, J = 2.1 Hz, H-2'), 7.65 (1H, dd, J = 8.5, 2.2 Hz, H-6'), 6.89 (1H, d, J = 8.4 Hz, H-5'), 6.41 (1H, d, J = 2.1 Hz, H-8), 6.22 (1H, d, J = 2.1 Hz, H-6), 5.13 (1H, d, J = 7.5 Hz, H-1''), 4.54 (1H, d, J = 1.7 Hz, H-1'''), 3.83 (1H, d, J = 10.8 Hz, H-6''), 3.66 (1H, dd, J = 3.5, 1.6 Hz, H-2'''), 3.56 (1H, dd, J = 9.5, 3.4 Hz, H-3'''), 1.14 (3H, d, J = 6.2 Hz, H-6''');^{13}CNMR (101 MHz, CD_3OD): δ_C 17.9 (C-6'''), 68.5 (C-6''), 69.7 (C-5'''), 71.4 (C-4''), 72.1 (C-2'''), 72.2 (C-3'''), 73.9 (C-4'''), 75.7 (C-2''), 77.2 (C-5''), 78.2 (C-3''), 94.9 (C-8), 99.9 (C-6), 102.4 (C-1'''), 104.7 (C-1''), 105.6 (C-10), 116.0 (C-5'), 117.7 (C-2'), 123.1 (C-1''), 123.6 (C-6'), 135.6 (C-3), 145.8 (C-3'), 149.8 (C-4'), 158.5 (C-9), 159.3 (C-2), 162.9 (C-5), 166.0 (C-7), 179.4 (C-4).

protocatechualdehyde (**11**): Yellow powder; *m/z* 139.0388 $[M + H]^+$ (calcd for $C_7H_7O_3$, 139.0390). ^1H NMR (400 MHz, CD_3OD): δ_H 7.30 (2H, dd, J = 4.3, 2.4 Hz, H-2, 6), 6.91 (1H, d, J = 8.6 Hz, H-5), 9.68 (1H, s, -CHO); ^{13}C NMR (101 MHz, CD_3OD): δ_C 130.8 (C-1), 115.3 (C-2), 147.1 (C-3), 153.7 (C-4), 116.2 (C-5), 126.4 (C-6), 193.1 (C-7).

caffeic acid (**12**): Yellow powder; *m/z* 181.0493 [M + H]$^+$ (calcd for $C_9H_9O_4$, 181.0495). ^{1}H NMR (400 MHz, CD_3OD) δ_H 7.04 (1H, d, *J* = 2.1 Hz, H-2), 6.78 (1H, d, *J* = 8.2 Hz, H-5), 6.93 (1H, dd, *J* = 8.2, 2.1 Hz, H-6),7.53 (1H, d, *J* = 15.8 Hz, H-7), 6.22 (1H, d, *J* = 15.9 Hz, H-8); ^{13}C NMR (101 MHz, CD_3OD): δ_C 127.7 (C-1), 115.0 (C-2), 146.7 (C-3), 149.4 (C-4), 116.5 (C-5), 122.9 (C-6), 147.0 (C-7), 115.5 (C-8), 171.1 (C-9).

caffeic acid ethyl ester (**13**): Yellow powder; *m/z* 209.0812 [M + H]$^+$ (calcd for $C_{11}H_{13}O_4$, 209.0808). ^{1}H NMR (400 MHz, $CDCl_3$) δ_H 7.03 (1H, d, *J* = 2.0 Hz, H-2), 6.78 (1H, d, *J* = 8.2 Hz, H-5), 6.92 (1H, dd, *J* = 8.1, 2.0 Hz, H-6), 7.52 (1H, d, *J* = 15.8 Hz, H-7), 6.25 (1H, d, *J* = 15.9 Hz, H-8), 4.20 (2H, q, *J* = 7.1 Hz, H-10), 4.60 (3H, t, *J* = 7.1 Hz, H-11); ^{13}C NMR (101 MHz, $CDCl_3$): δ_C 127.7 (C-1), 115.3 (C-2), 146.7 (C-3),149.5 (C-4), 116.5 (C-5),122.9 (C-6), 146.7 (C-7), 115.1 (C-8), 169.3 (C-9), 61.5 (C-10), 14.6 (C-11).

caffeic acid ethylene ester (**14**): Yellow powder; *m/z* 205.0504 [M + H]$^+$ (calcd for $C_{11}H_9O_4$, 205.0506). ^{1}H NMR (400 MHz, $CDCl_3$) δ_H 7.07 (1H, d, *J* = 2.0 Hz, H-2), 6.78 (1H, d, *J* = 8.3 Hz, H-5), 6.97 (1H, dd, *J* = 8.3, 2.0 Hz, H-6), 7.64 (1H, d, *J* = 15.9 Hz, H-7), 6.25 (1H, d, *J* = 15.9 Hz, H-8), 7.38 (1H, dd, *J* = 14.0, 6.3 Hz, H-10), 4.93 (1H, dd, *J* = 14.0, 1.4 Hz, H-11*α*), 4.60 (1H, dd, *J* = 6.3, 1.5 Hz, H-11*β*); ^{13}C NMR (101 MHz, $CDCl_3$): δ_C127.5 (C-1), 115.3 (C-2), 146.8 (C-3), 150.0 (C-4), 116.6 (C-5), 123.4 (C-6), 148.7 (C-7), 113.5 (C-8), 165.5 (C-9), 142.5 (C-10), 97.7 (C-11).

salicylic acid (**15**): Colorless crystal; *m/z* 137.0247 [M - H]$^-$ (calcd for $C_7H_5O_3$, 137.0244). ^{1}H NMR (400 MHz, CD_3OD) : δ_H 7.85 (1H, dd, *J* = 7.9, 1.8 Hz, H-2), 7.39 (1H, ddd, *J* = 8.5, 7.2, 1.5 Hz, H-3), 6.92-6.81 (2H, m, H-4/5); ^{13}C NMR (101 MHz, CD_3OD) : δ_C 112.9 (C-4), 117.8 (C-5), 119.7 (C-6), 131.6 (C-3), 135.7 (C-1), 163.0 (C-2), 174.4 (C-7)

carvacrol (**16**):Yellow oil; ^{1}H NMR (400 MHz, $CDCl_3$): δ_H 7.04 (1H, d, *J* = 7.6 Hz, H-3), 6.73 (1H, dd, *J* = 7.6, 1.7 Hz, H-6), 6.66 (1H, d, *J* = 1.8 Hz, H-4), 2.82 (1H, tq, *J* = 13.5, 6.6 Hz, H-7), 2.22 (3H, s, H-10), 1.22 (6H, d, *J* = 6.9 Hz, H-8/9); ^{13}C NMR (101 MHz, $CDCl_3$): δ_C 15.4 (C-10), 24.1 (C-8、9), 33.8 (C-7), 113.1 (C-6), 118.9 (C-4), 120.9 (C-2), 130.9 (C-3), 148.6 (C-5), 153.8 (C-1).

thymol (**17**): White powder; ^{1}H NMR (400 MHz, $CDCl_3$) : δ_H 7.17 (1H, d, *J* = 7.7 Hz, H-5), 6.82 (1H, d, *J* = 7.8 Hz, H-6), 6.62 (1H, d, *J* = 1.8 Hz, H-2), 3.25 (1H, hept, *J* = 6.8 Hz, H-8), 2.34 (3H, s, H-7), 1.32 (6H, dd, *J* = 7.0, 1.5 Hz, H-9/10); ^{13}C NMR (101

MHz, $CDCl_3$): δ_C 20.9 (C-7), 22.8 (C-9、10), 26.8 (C-8), 116.2 (C-2), 121.8 (C-6), 126.3 (C-5), 131.6 (C-4), 136.7 (C-1), 152.6 (C-3).

β-sitosterol (**18**): White crystals; ^{1}H NMR (400 MHz, $CDCl_3$): δ_H 5.34 (1H, dd, *J* = 5.3, 2.5 Hz, H-6), 3.60-3.43 (1H, m, H-3), 2.33-2.17 (2H, m, H-4); ^{13}C NMR (101 MHz, $CDCl_3$): δ_C 36.3 (C-1), 71.9 (C-3), 42.4 (C-4), 140.9 (C-5),32.0 (C-6), 32.0 (C-7), 50.3 (C-8), 37.4 (C-9), 23.2 (C-10), 40.6 (C-11), 42.5 (C-12), 57.0 (C-13), 25.5 (C-14), 56.2 (C-15), 36.6 (C-17), 29.3 (C-18), 46.0 (C-21), 29.1 (C-22), 20.0 (C-23), 19.2 (C-24), 12.0 (C-25), 19.5 (C-26), 24.5 (C-27), 12.1 (C-28), 21.2 (C-29).

stigmasta-4,22-dien-3-one (**19**): White crystals; *m/z* 411.3629 $[M + H]^+$ (calcd for $C_{29}H_{47}O$, 411.3621). ^{1}H NMR (400 MHz, $CDCl_3$): δ_H 5.67 (1H, d, *J* = 1.8 Hz, H-4), 5.10 (1H, dd, *J* = 15.2, 8.5 Hz, H-23), 4.97 (1H, dd, *J* = 15.2, 8.6 Hz, H-22), 1.13 (3H, s, H-19), 0.97 (3H, d, *J* = 6.6 Hz, H-21), 0.87-0.84 (3H, m, H-26), 0.79 (3H, d, *J* = 2.4 Hz, H-27), 0.77 (3H, d, *J* = 1.8 Hz, H-29), 0.76 (3H, d, *J* = 2.3 Hz, H-18); ^{13}C NMR (151 MHz, $CDCl_3$): δ_C 199.9 (C-3), 171.9 (C-5), 138.3 (C-22), 129.5 (C-23), 123.9 (C-4), 56.1 (C-14), 56.0 (C-17), 53.9 (C-9), 51.3 (C-24), 42.4 (C-13), 40.6 (C-20), 39.6 (C-12), 38.7 (C-10), 35.8 (C-8), 35.7 (C-1), 34.1 (C-2), 33.1 (C-6), 32.1 (C-7), 32.0 (C-25), 29.0 (C-16), 25.5 (C-28), 24.4 (C-15), 21.3 (C-26), 21.2 (C-21), 21.1 (C-11), 19.1 (C-27), 17.5 (C-19), 12.4 (C-29), 12.3 (C-18).

stigmasta-4,22-dien-3,6-dione (**20**): White crystals; *m/z* 425.3418 $[M + H]^+$ (calcd for $C_{29}H_{45}O_2$, 425.3414). ^{1}H NMR (400 MHz, $CDCl_3$): δ_H 6.15 (1H, d, *J* = 0.9 Hz, H-4), 5.14 (1H, dd, *J* = 15.2, 8.5 Hz, H-23), 5.03 (1H, dd, *J* = 15.2, 8.6 Hz, H-22), 1.15 (3H, s, H-19), 1.02 (3H, d, *J* = 6.6 Hz, H-21), 0.73 (3H, s, H-18); ^{13}C NMR (101 MHz, $CDCl_3$): δ_C 202.4 (C-3), 199.5 (C-6), 161.1 (C-5), 137.9 (C-22), 129.9 (C-23), 125.6 (C-4), 56.8 (C-14), 56.0 (C-17), 51.3 (C-24), 46.9 (C-7), 42.5 (C-13), 39.9 (C-20), 39.2 (C-10), 36.1 (C-1), 34.3 (C-8), 32.5 (C-25), 28.8 (C-16), 25.5 (C-28), 24.1 (C-15), 21.3 (C-27), 21.0 (C-21), 19.1 (C-26), 19.1 (C-11), 17.6 (C-19), 12.1 (C-29), 12.0 (C-18).

stigmasta-5,22-dien-3,7-dione (**21**): White crystals; *m/z* 425.3418 $[M + H]^+$ (calcd for $C_{29}H_{45}O_2$, 425.3414). ^{1}H NMR (400 MHz, $CDCl_3$): δ_H 6.15 (1H, d, *J* = 0.9 Hz, H-6), 5.14 (1H, dd, *J* = 15.2, 8.5 Hz, H-22), 5.03 (1H, dd, *J* = 15.2, 8.6 Hz, H-23); ^{13}C NMR (101 MHz, $CDCl_3$): δ_C 202.4 (C-7), 199.6 (C-3), 161.2 (C-5), 137.9 (C-22), 129.9 (C-23), 125.5 (C-6), 56.7 (C-17), 55.9 (C-14), 51.1 (C-9), 51.1 (C-24), 46.9 (C-8), 45.9 (C-4), 42.6 (C-13), 39.9 (C-20), 39.2 (C-10), 39.1 (C-1), 35.6 (C-12), 34.1 (C-2),

32.0 (C-25), 29.3 (C-16), 26.2 (C-15), 24.1 (C-11), 23.2 (C-28), 21.2 (C-21), 21.0 (C-26), 19.9 (C-27), 17.6 (C-19), 12.4 (C-29), 12.2 (C-18).

ursolic acid (**22**): White powder; *m/z* 457.3672 $[M + H]^+$ (calcd for $C_{30}H_{49}O_3$, 457.3676). ^1H NMR (400 MHz, $CDCl_3$): δ_H 5.05 (1H, t, J = 3.4 Hz, H-12), 3.16 (1H, dd, J = 11.1, 5.2 Hz, H-3), 1.00 (3H, s, H-26), 0.93 (6H, d, J = 4.5 Hz, H-23/30), 0.88 (3H, s, H-29), 0.80 (3H, d, J = 7.5 Hz, H-27), 0.72 (6H, d, J = 2.8 Hz, H-24/25); ^{13}C NMR (101 MHz, $CDCl_3$): δ_C 14.3 (C-25), 15.8 (C-24), 17.0 (C-29), 17.6 (C-26), 18.5 (C-6), 21.5 (C-30), 22.8 (C-27), 23.4 (C-11), 24.9 (C-16), 26.8 (C-2), 27.4 (C-15), 28.3 (C-23), 31.4 (C-21), 33.1 (C-7), 37.0 (C-10), 38.9 (C-1、4), 39.8 (C-8、19), 41.7 (C-14), 47.9 (C-17), 55.3 (C-5), 59.2 (C-18), 79.3 (C-3), 124.6 (C-12), 139.7 (C-13), 178.8 (C-28).

ursolic aldehyde (**23**): White powder; *m/z* 463.3555 $[M + Na]^+$ (calcd for $C_{30}H_{48}NaO_2$, 463.3547). ^1H NMR (400 MHz, $CDCl_3$): δ_H 9.30 (1H, d, J = 1.3 Hz, H-28), 5.29 (1H, t, J = 3.8 Hz, H-12), 3.19 (1H, dd, J = 10.7, 5.1 Hz, H-3), 1.07 (3H, s, H-27), 0.96 (3H, s, H-23), 0.94 (3H, d, J = 2.5 Hz, H-30), 0.90 (3H, s, H-25), 0.85 (3H, d, J = 6.5 Hz, H-29), 0.76 (3H, s, H-24), 0.74 (3H, s, H-26); ^{13}C NMR (101 MHz, $CDCl_3$): δ_C 15.6 (C-25), 15.7 (C-24), 16.8 (C-29), 17.3 (C-26), 18.4 (C-6), 21.2 (C-30), 23.3 (C-11), 23.4 (C-16), 27.0 (C-15), 27.3 (C-2), 28.3 (C-23), 30.3 (C-21), 32.0 (C-7), 33.2 (C-22), 37.0 (C-10), 38.8 (C-1, 4), 38.9 (C-20), 39.1 (C-19), 39.9 (C-8), 42.3 (C-14), 47.7 (C-9), 50.2 (C-17), 52.7 (C-18), 55.3 (C-5), 79.1 (C-3), 126.3 (C-12), 137.9 (C-13), 207.6 (C-28).

maslinic acid (**24**): Yellow powder; *m/z* 495.3450 $[M + Na]^+$ (calcd for $C_{30}H_{48}NaO_4$, 495.3445). ^1H NMR (400 MHz, DMSO-d_6): δ_H 12.01 (1H, s, -COOH), 5.17 (1H, d, J = 3.6 Hz, H-12), 4.32 (1H, d, H-2), 2.74 (1H, d, J = 8.9 Hz, H-3), 1.09 (3H, s, H-27), 0.92 (3H, s, H-25), 0.90 (3H, s, H-26), 0.87 (6H, s, H-29/30), 0.70 (6H, d, J = 2.2 Hz, H-23/24); ^{13}C NMR (101 MHz, DMSO-d_6): δ_C 16.3 (C-26), 16.9 (C-25), 17.1 (C-24), 18.1 (C-6), 22.6 (C-11), 23.0 (C-16), 23.4 (C-30), 25.7 (C-27), 27.2 (C-15), 28.8 (C-23), 30.4 (C-20), 32.1 (C-22), 32.3 (C-7), 32.9 (C-29), 33.3 (C-21), 37.7 (C-10), 38.9 (C-4), 38.9 (C-8), 40.8 (C-18), 41.3 (C-14), 45.4 (C-17), 45.7 (C-19), 46.8 (C-1), 47.1 (C-9), 54.8 (C-5), 67.1 (C-2), 82.2 (C-3), 121.5 (C-12), 143.9 (C-13), 178.6 (C-28).

tormentic acid (**25**):Yellowish-brown powder; *m/z* 487.3426 $[M - H]^-$ (calcd for $C_{30}H_{47}O_5$, 487.3429). ^1H NMR (400 MHz, DMSO-d_6): δ_H 5.17 (1H, s, H-12), 3.75 (1H, d, J = 5.7 Hz, 19-OH), 2.74 (1H, d, J = 9.4 Hz, H-3), 2.37 (1H, s, H-18), 1.28

(3H, d, J = 4.0 Hz, H-27), 1.08 (3H, s, H-29), 0.92 (6H, d, J = 7.1 Hz, H-25/26), 0.85 (3H, d, J = 5.3 Hz, H-30), 0.70 (6H, d, J = 4.6 Hz, H-23/24); ^{13}C NMR (101 MHz, DMSO-d_6): δ_C 16.3 (C-15、30), 16.6 (C-26), 17.2 (C-24), 18.2 (C-6), 23.2 (C-11), 24.0 (C-27), 25.2 (C-16), 26.0 (C-21), 26.4 (C-29), 28.1(C-15), 28.8 (C-23), 32.6 (C-7), 37.3 (C-10), 37.6 (C-22), 39.0 (C-4), 39.4 (C-8), 41.2 (C-14), 41.4 (C-20), 46.7 (C-9), 46.9 (C-1), 47.0 (C-17), 53.2 (C-18), 54.9 (C-5), 67.2 (C-2), 71.7 (C-19), 82.3 (C-3), 126.8 (C-12), 138.7 (C-13), 179.0 (C-28).

α-cedrene (**26**): Light yellow liquid; ^{1}H NMR (400 MHz, $CDCl_3$): δ_H 5.23 (1H, dq, J = 3.9, 1.8 Hz, H-4), 2.18 (1H, dp, J = 16.7, 2.5 Hz, H-5), 1.68 (3H, q, J = 2.0 Hz, H-15), 1.03 (3H, s, H-13), 0.96 (3H, s, H-12), 0.85 (3H, d, J = 7.2 Hz, H-14); ^{13}C NMR (101 MHz, $CDCl_3$): δ_C 15.6 (C-14), 24.9 (C-15), 25.0 (C-9), 25.8 (C-12), 27.8 (C-13), 36.3 (C-8), 39.0 (C-5), 40.8 (C-1), 41.6 (C-7), 48.3 (C-11), 54.0 (C-6), 55.0 (C-2), 59.2 (C-10), 119.4 (C-4), 140.7 (C-3).

clovane-2,9-diol (**27**): Yellow oil; m/z 256.2270 $[M + NH_4]^+$ (calcd for $C_{15}H_{30}NO_2$, 256.2271). ^{1}H NMR (400 MHz, CD_3OD) : δ_H 3.73 (1H, dd, J = 10.6, 5.9 Hz, H-11), 3.23 (1H, d, J = 3.0 Hz, H-4), 1.99 (1H, tdd, J = 14.2, 5.0, 3.2 Hz, H-7), 1.71 (1H, dd, J = 13.4, 4.7 Hz, H-3), 1.03 (3H, s, H-14), 0.93 (3H, s, H-12), 0.86 (3H, s, H-15); ^{13}C NMR (101 MHz, CD_3OD): δ_C 21.7 (C-13), 25.8 (C-15), 26.9 (C-7), 27.8 (C-3), 29.1 (C-12), 31.8 (C-14), 34.5 (C-6), 35.8 (C-5), 36.7 (C-2), 37.7 (C-9), 45.4 (C-1), 48.2 (C-10), 52.0 (C-8), 75.9 (C-4), 81.4 (C-11).

α-cyperone (**28**): Yellow oil; m/z 219.1750 $[M + H]^+$ (calcd for $C_{15}H_{23}O$, 219.1743). ^{1}H NMR (400 MHz, $CDCl_3$): δ_H 4.77 (2H, d, J = 1.3 Hz, H-12), 2.79-2.67 (1H, m, H-8a), 2.52 (1H, ddd, J = 16.9, 13.4, 6.1 Hz, H-3α), 2.41 (1H, t, J = 4.1 Hz, H-3β), 2.10-1.98 (1H, m, H-8β), 1.77 (3H, q, J = 1.1 Hz, H-13), 1.22 (3H, s, H-9); ^{13}C NMR (101 MHz, $CDCl_3$): δ_C 11.1 (C-10), 20.8 (C-13), 22.7 (C-9), 27.0 (C-6), 33.1 (C-8), 34.0 (C-3), 36.0 (C-15), 37.6(C-4), 42.1 (C-5), 46.1 (C-7), 109.3 (C-12), 129.0 (C-1), 149.3 (C-11), 162.3 (C-14), 199.3 (C-2).

esculetin (**29**): Yellow powder; m/z 177.0915 $[M - H]^-$(calcd for $C_9H_5O_4$, 177.0193). ^{1}H NMR (400 MHz, DMSO-d_6): δ_H 7.84 (1H, d, J = 9.4 Hz, H-4), 6.97 (s, 1H, H-5), 6.74 (1H, s, H-8), 6.15 (1H, d, J = 9.4 Hz, H-3); ^{13}C NMR (101 MHz, DMSO-d_6): δ_C 102.7 (C-5), 110.8 (C-8), 111.6 (C-3), 112.4 (C-9), 142.9 (C-7), 144.5 (C-4), 148.6 (C-6), 150.5 (C-10), 160.9 (C-2).

gusanlung C (**30**): Yellow powder; m/z 314.1396 $[M + H]^+$ (calcd for $C_{18}H_{20}NO_4$, 314.1387). ^{1}H NMR (400 MHz, Acetone-d_6): δ_H 7.15 (1H, d, J = 1.9 Hz, H-1), 7.03

(1H, d, J = 1.9 Hz, H-3), 6.83 (1H, d, J = 8.1 Hz, H-4), 2.75 (2H, t, J = 7.3 Hz, H-5), 3.49 (2H, t, J = 7.4 Hz, H-6), 7.06 (1H, d, J = 8.4 Hz, H-9), 6.76 (1H, d, J = 8.4 Hz, H-10), 6.76 (1H, d, J = 8.4 Hz, H-12), 7.06 (1H, d, J = 8.4 Hz, H-13), 7.46 (1H, dd, J = 15.6, 2.8 Hz, H-15), 6.51 (1H, d, J = 15.6 Hz, H-16), 3.87 (3H, s, -OCH3); ^{13}C NMR (101 MHz, Acetone-d_6): δ_C 120.0 (C-1), 156.6 (C-2), 122.6 (C-3), 116.0 (C-4), 131.1 (C-4a), 35.7 (C-5), 41.8 (C-6), 166.5 (C-8), 116.0 (C-9), 130.5 (C-10), 149.2 (C-11), 130.5 (C-12), 116.0 (C-13), 148.6 (C-14), 140.4 (C-15), 111.3 (C-16), 128.3 (C-16a), 56.2 (-OCH_3).

(1*R*,2*R*,2'*E*)-2-[5'-(hydroxy)-2-penten-1-yl]-3-oxocyclopentane acetic acid methyl ester (**31**): White powder; *m/z* 241.1434 $[M + H]^+$ (calcd for $C_{13}H_{21}O_4$, 241.1434). ^{1}H NMR (400 MHz, CD_3OD): δ_H 2.38 (1H, m, H-1), 1.99 (1H, m, H-2), 2.10 (1H, m, H-4), 2.20 (1H, m, H-5α), 1.53 (1H, m, H-5β), 2.35 (1H, m, H-1'), 5.42 (1H, m, H-2'), 5.49 (1H, m, H-3'), 2.25 (1H, m, H-4'), 3.55 (1H, t, J = 6.8 Hz, H-5'), 2.72 (1H, t, J =14.8, 4.0 Hz, H-1''α), 2.36 (1H, m, H-1''β), 3.68 (3H, s, H-3''); ^{13}C NMR (101 MHz, CD_3OD): δ_C 39.1 (C-1), 55.0 (C-2),38.5 (C-4), 28.0 (C-5), 26.4 (C-1'), 129.0 (C-2'), 129.1 (C-3'), 31.8 (C-4'), 62.5 (C-5'), 39.5 (C-1''), 174.4 (C-2''), 52.1 (C-3'')

vomifoliol (**32**): Yellow powder; *m/z* 314.1396 $[M + H]^+$ (calcd for $C_{18}H_{20}NO_4$, 314.1387). ^{1}H NMR (400 MHz, $CDCl_3$): δ_H 2.43 (1H, d, J = 17.1 Hz, H-2α), 2.22 (1H, d, J = 17.1 Hz, H-2β), 5.88 (1H, s, H-4), 5.76 (1H, d, J = 15.7 Hz, H-7), 5.82 (1H, d, J = 5.1 Hz, H-8), 4.38 (1H, p, J = 6.2 Hz, H-9), 1.28 (3H, d, J = 6.4 Hz, H-10), 0.99 (3H, d, J = 4.5 Hz, H-11), 1.07 (3H, d, J = 6.7 Hz, H-12), 1.89 (3H, s, H-13); ^{13}C NMR (101 MHz, $CDCl_3$): δ_C 41.3 (C-1), 49.8 (C-2), 198.5 (C-3), 129.0 (C-4), 163.4 (C-5), 79.1 (C-6), 126.9 (C-7), 135.9 (C-8), 68.0 (C-9), 23.0 (C-10), 24.2 (C-11), 23.0 (C-12), 19.1 (C-13)

m-cymene (**33**): Colorless crystals; ^{1}H NMR (400 MHz, CD_3OD): δ_H 7.11 (1H, t, J = 7.6 Hz, H-5), 7.01 (1H, s, H-2), 6.97 (1H, d, J = 7.8 Hz, H-6), 6.94 (1H, d, J = 7.6 Hz, H-4), 2.81 (1H, hept, J = 6.9 Hz, H-7), 2.28 (3H, s, H-8), 1.21 (6H, d, J = 7.0 Hz, H-9, 10); ^{13}C NMR (101 MHz, CD_3OD): δ_C 21.6 (C-8), 24.5 (C-9、10), 35.3 (C-7), 124.3 (C-6), 127.4 (C-4), 128.0 (C-2), 129.2 (C-5), 138.7 (C-3), 149.9 (C-1).

5-hydroxymethylfurfural (**34**): Light yellow crystals; *m/z* 127.0393 $[M + H]^+$ (calcd for $C_6H_7O_3$, 127.0390). ^{1}H NMR (400 MHz, $CDCl_3$): δ_H 9.55 (1H, s, -CHO), 7.20 (1H, d, J = 3.6 Hz, H-3), 6.50 (1H, d, J = 3.6 Hz, H-4), 4.69 (2H, s, -CH_2OH); ^{13}C NMR (101 MHz, $CDCl_3$): δ_C 152.4 (C-2), 123.1 (C-3), 110.1 (C-4), 160.9 (C-5), 177.8 (-CHO), 57.6 (-CH_2OH)

cineole (**35**): Colorless liquid; ^{1}H NMR (400 MHz, $CDCl_3$): δ_H 2.06-1.96 (2H, m, H-2), 1.49 (4H, dd, J = 8.8, 1.3 Hz, H-3, 5), 1.23 (s, 6H, H-9、10), 1.04 (s, 3H, H-7); ^{13}C NMR (101 MHz, $CDCl_3$): δ_C 23.0 (C-9、10), 27.7 (C-7), 29.0 (C-2, 6), 31.6 (C-3, 5), 33.1 (C-4), 70.0 (C-1), 73.8 (C-8)

azelaic acid (**36**): White powder; m/z 189.1124 $[M + H]^+$ (calcd for $C_9H_{17}O_4$, 189.1121). ^{1}H NMR (400 MHz, CD_3OD): δ_H 2.28 (4H, t, J= 7.3 Hz, H-2, 8), 1.60 (4H, t, J = 8.5 Hz, H-3, 7), 1.34 (6H, m, H-4, 5, 6), ^{13}C NMR (101 MHz, CD_3OD): δ_C 177.6 (C-2), 34.9 (C-3), 26.0 (C-4), 30.0 (C-5), 30.0 (C-6), 30.0 (C-7), 34.9 (C-8), 177.6 (C-9).

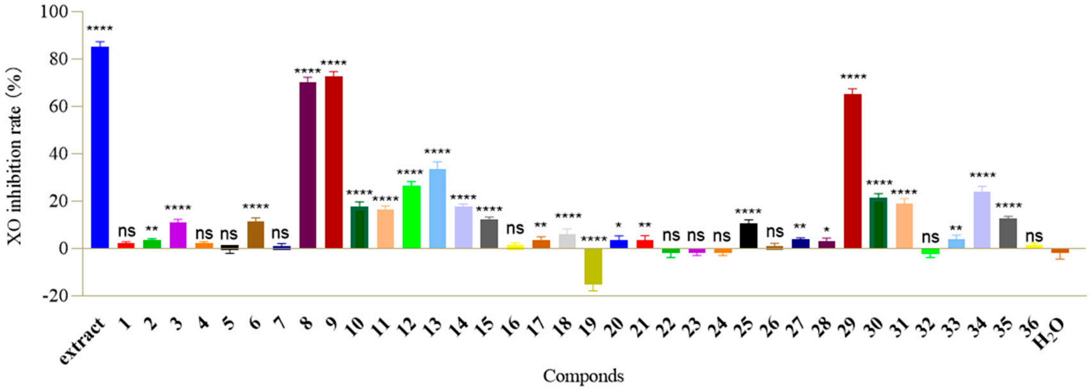

Figure S19 XO inhibitory activity of compounds compounds **1-36** and *C. strobilifer* extract at 0.2 mM. (n = 3, ns = nonsignificance, * $p < 0.01$, ** $p < 0.001$, **** $p < 0.00001$ compared to H_2O).

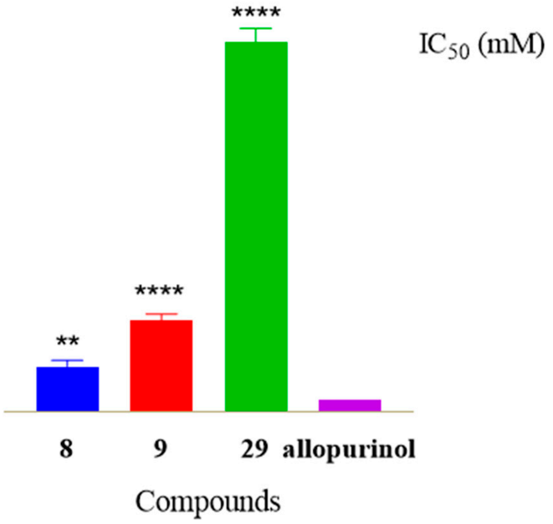

Figure S20 XO inhibitory activity of compounds **8**, **9**, **29** and allopurinoln (n = 3; allopurinol was used as a positive control). (** $p < 0.001$, **** $p < 0.00001$ compared to allopurinol)

Table S1 XO inhibitory activity of compounds **1-36** and allopurinol

Compound	IC_{50} (mM)[a]
**8**	0.034 ± 0.004
**9**	0.067 ± 0.005
**29**	0.284 ± 0.01
others	＞ 0.3
Allopurinol[b]	0.009 ± 0.000117

[a] IC_{50} values were presented as means ± SD (n = 3). [b] Allopurinol was used as a positive control.

Determination of the content of compounds 8, 9, and 29 in *Coleus strobilife*r

Preparation of Samples

*Coleus strobilife*r (50 g) underwent dual reflux extractions with water and 70% ethanol (8× and 6× solvent volumes for 2 h and 1 h, respectively). Combined filtrates were concentrated *via* rotary evaporation and lyophilized to yield aqueous/ethanolic extracts. For analysis, 200 mg of extract was dissolved in methanol (10 mL volumetric flask), sonicated (30 min), adjusted to volume, filtered (0.22 μm), and the filtrate retained.

Preparation of Standard Solutions

An appropriate amount of apigenin (**8**), luteolin (**9**), and esculetin (**29**) reference standards was precisely weighed and dissolved in methanol to prepare a mixed standard solution containing 1 mg/mL of each compound.

High-performance liquid chromatography

This research was carried on a Waters UPLC system comprising a PDA detector. The chromatographic analysis was performed using an ACQUITY UPLC® HSS T3 column (2.1 mm × 100 mm, 1.8 μm) maintained at 30°C. The mobile phase comprised two components: (A) aqueous solution containing 0.2% acetic acid and (B) acetonitrile. Separation was achieved at a flow rate of 0.4 mL/min with UV detection

monitored at 340 nm. Samples were injected at a volume of 1 μL using the following optimized gradient elution protocol: an elution ratio of 0-2 min, acetonitrile (10%~10%); 2-9 min, acetonitrile (10%~20%); 9-28 min, acetonitrile (20%~30%); 28-38 min, acetonitrile (30%~90%); 38-40 min, acetonitrile (90%~10%).

Establishment of the standard curve

The standard stock solution prepared above was serially diluted with methanol to obtain working solutions at concentrations of 0.625, 1.25, 2.5, 5, 10, and 20 μg/mL. A linear calibration curve was constructed by plotting the peak areas against the corresponding concentrations of the injected solutions, and the regression equation was subsequently derived through statistical analysis.

Table S2 Analytical figures of merit by UPLC.

Analyte	Calibration relationship	Correlation coefficient	Linear dynamic range (μg/mL)
**8**	Y=8884X-3832.8	0.9996	0.625~20
**9**	Y=7040.1X-4366.6	0.9985	0.625~20
**29**	Y=3424X-1137.4	0.9998	0.625~20

Result

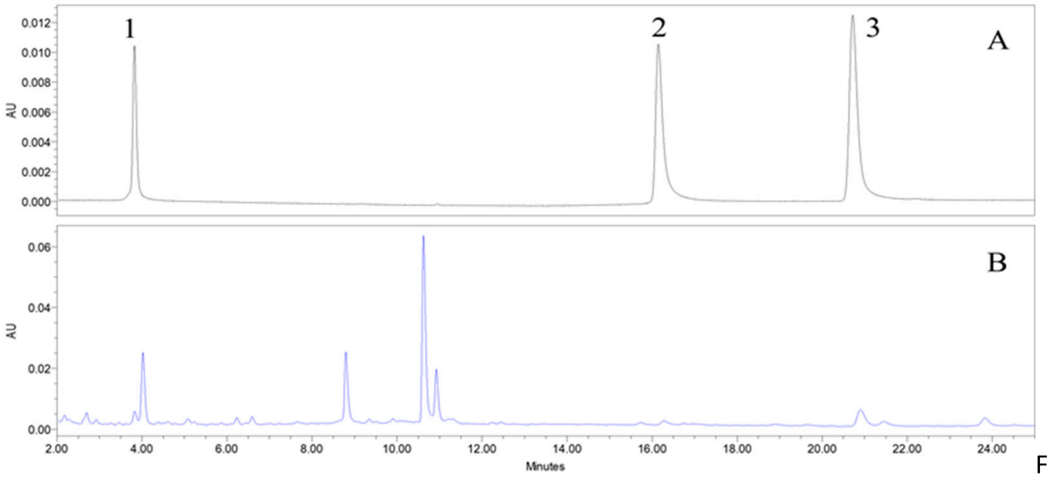

Figure

Figure S21 UPLC of standards and samples (A- standard solution, B- sample solution, 1- esculetin (**29**), 2- luteolin (**9**), 3- apigenin (**8**)).

Table S3 The contents of **8**, **9**, and **29** in *C. strobilife*r extract (n = 2).

Samples	Content (**8**)	Content (**9**)	Content (**29**)
Water extract of *C. strobilife*r-1	/	/	0.0385%
Ethanol extract of *C. strobilife*r-1	0.0065%	0.0207%	0.0645%
Water extract of *C. strobilife*r-2	0.0098%	0.0048%	0.0276%
Ethanol extract of *C. strobilife*r-2	0.0397%	0.0121%	0.0272%

Table S4 XOD inhibitory activity of compounds **8**, **9**, **29**, and *C. strobilife*r extract.

Samples	Content (μg/mL)	XO inhibition rate
*C. strobilife*r extract	1000	90.1%
**8**	0.397	8.4%
**9**	0.207	5.3%
**29**	0.645	3.2%
Mixed solutions of **8**, **9** and **29**	0.645+0.207+0.397	10.8%
